# Efficacy and safety of consolidation durvalumab after chemoradiation therapy for stage III non-small-cell lung cancer: a systematic review, meta-analysis, and meta-regression of real-world studies

**DOI:** 10.3389/fphar.2023.1103927

**Published:** 2023-06-08

**Authors:** Yatong Zhang, Yumei Tian, Li Zheng, Xuelin Sun, Zinan Zhao, Yujing Zheng, Jinhui Tian

**Affiliations:** ^1^ Evidence-Based Medicine Center, School of Basic Medical Sciences, Lanzhou University, Lanzhou, China; ^2^ Department of Pharmacy, Beijing Hospital, Beijing, China; ^3^ National Center of Gerontology, Institute of Geriatric Medicine, Chinese Academy of Medical Sciences, Beijing, China; ^4^ Beijing Key Laboratory of Assessment of Clinical Drugs Risk and Individual Application (Beijing Hospital), Beijing, China; ^5^ School of Nursing, Hunan University of Medicine, Huaihua, Hunan, China; ^6^ Department of Pharmacy, China Aerospace Science and Industry Corporation 731 Hospital, Beijing, China

**Keywords:** immunotherapy, immune checkpoint inhibitors, survival, pneumonitis, real-world, lung cancer

## Abstract

**Background:** The current review aimed to pool real-world evidence on the efficacy and toxicity of consolidation durvalumab for stage III unresectable non-small cell lung cancer (NSCLC) after curative chemoradiotherapy.

**Methods:** PubMed, CENTRAL, ScienceDirect, Embase, and Google Scholar were searched for observational studies reporting the use of durvalumab for NSCLC till 12th April 2022. Twenty-three studies with 4,400 patients were included.

**Results:** The pooled 1-year overall survival (OS) and progression-free survival rates (PFS) were 85% (95% CI: 81%–89%) and 60% (95% CI: 56%–64%) respectively. Pooled incidence of all-grade pneumonitis, grade ≥3 pneumonitis and discontinuation of durvalumab due to pneumonitis were 27% (95% CI: 19%–36%), 8% (95% CI: 6%–10%) and 17% (95% CI: 12%–23%) respectively. The pooled proportion of patients experiencing endocrine, cutaneous, musculoskeletal, and gastrointestinal adverse events was 11% (95% CI: 7%–18%), 8% (95% CI: 3%–17%), 5% (95% CI: 3%–6%), and 6% (95% CI: 3%–12%), respectively.

**Conclusion:** Meta-regression indicated that performance status significantly influenced PFS, while age, time to durvalumab, and programmed death-ligand 1 status significantly affected pneumonitis rates. Real-world evidence suggests that the short-term efficacy and safety of durvalumab are consistent with that of the PACIFIC trial. The congruence of results lends support to durvalumab use in improving outcomes of unresectable stage III NSCLC.

**Systematic Review Registration:** https://www.crd.york.ac.uk/prospero/display_record.php?ID=CRD42022324663, identifier CRD42022324663.

## Introduction

Lung cancer is one of the leading causes of cancer-related death worldwide (Bray et al., 2018). Non-small cell lung cancer (NSCLC) represents about 80% of all lung cancer cases. Over 30% of NSCLC cases are detected in a locally-advanced stage which is often unresectable (Siegel et al., 2012). Around 25% of NSCLC patients are diagnosed as stage III disease. This heterogeneous group of patients is primarily treated with curative platinum-based chemoradiotherapy (CRT) regardless of histology and molecular subtype (Xiao and Hong, 2021). While concurrent CRT (cCRT) improves survival rates, the prognosis of stage III NSCLC remains poor, with a median progression-free survival (PFS) of 8 months and a 5-year overall survival (OS) of 15%–30% (Yoon et al., 2017).

The PACIFIC trial (Antonia et al., 2017) that aimed to explore better therapeutic options for NSCLC, has provided a new standard of management of unresectable stage III NSCLC patients. This phase 3 randomized controlled trial (RCT) examined the efficacy of consolidation durvalumab, a programmed death-ligand 1 (PD-L1) immune checkpoint inhibitor (ICI), following curative cCRT for stage III disease. Recently released 5-year results of the trial indicate an OS of 42.9% and PFS of 33.1% with consolidation durvalumab therapy (Faivre-Finn et al., 2021). These outcomes have validated the survival benefits offered by durvalumab as compared to CRT alone thereby establishing a new standard-of-care. Durvalumab has now been approved by healthcare authorities around the globe for the management of unresectable stage III disease (Ettinger et al., 2019).

It is well-known that patients enrolled in clinical trials are not entirely representative of patients seen in clinical practice. The rigorous inclusion criteria of RCTs often exclude the elderly, those with comorbidities, and with poor performance status leading to the inclusion of a younger and healthier patient population who may endure treatment-related adverse events with clinical benefits (Blonde et al., 2018). Therefore, the results of pivotal RCTs often lack external validity and need to be complemented by real-world data that include heterogeneous patient population (Pasello et al., 2020), and may provide more detailed information on the actual efficacy and toxicity of the new therapeutic agent. Since the results of the PACIFIC trial (Antonia et al., 2017), several studies have reported the real-world safety and efficacy of consolidation durvalumab for stage III NSCLC after curative CRT (Faehling et al., 2020; Jung et al., 2020; Miura et al., 2020; Desilets et al., 2021). Recently, a systematic review by Wang et al. (2022) aimed to collate such evidence. However, this review included just 13 studies. Furthermore, the survival analysis of the review had only five studies and a meta-regression analysis was not performed due to insufficient data. In view of these limitations, the aim of our current study was to conduct an updated systematic review and meta-analysis assessing the real-world efficacy and safety of consolidation durvalumab after CRT for stage III NSCLC.

## Material and methods

### Search and eligibility

The protocol of the study was pre-registered in the online database PROSPERO (No CRD42022324663). The reporting guidelines of PRISMA were followed (Page et al., 2021). A literature search was conducted in the online databases such as PubMed, CENTRAL, ScienceDirect, Embase, and Google Scholar for English-language observational studies reporting the use of durvalumab for NSCLC. The search was initiated from 1^st^ January 2017 and completed on 12th April 2022. The following broad search terms were used: “lung cancer”, “lung carcinoma,” “NSCLC,” and “durvalumab.” The search string used was {[(lung cancer) OR (lung carcinoma)] OR (NSCLC)} AND (durvalumab). The search results were consolidated and deduplicated for the initial screening of titles and abstracts. Relevant studies were then extracted and checked for eligibility. The eligibility criteria were defined as: 1) Observational studies reporting the use of consolidation durvalumab for unresectable stage III NSCLC after curative CRT. 2) Studies reporting data on OS, PFS, or adverse events. We excluded studies on early-stage NSCLC, combining CRT with surgery, using palliative therapy, tyrosine kinase inhibitors, or other ICIs instead of durvalumab. Studies with duplicate data, clinical trials, review articles, and case reports were also excluded. Two review authors carried out the literature search, and independently performed initial and final screening of the studies. In the final stage, the full-text articles were screened based on the eligibility criteria, and only studies meeting the criteria were included. Any differences in the selection process were resolved by consulting the third reviewer. Lastly, the bibliography of the included studies and previous reviews was hand-searched to check for any missed relevant studies.

### Data management

Data from the eligible studies were extracted using an Excel spreadsheet and included the following: study authors, publication year, study location, number of centers, sample size, median age, Eastern Cooperative Oncology Group performance status (ECOG PS) score, type of CRT, the dose of radiotherapy, chemotherapy regimen, time of durvalumab administration after CRT, durvalumab treatment duration, PD-L1 status, driver gene mutation status, and outcome data.

The primary outcome data included 1-year OS, 1-year PFS, and incidence of all-grade pneumonitis. Secondary outcomes included incidence of grade ≥3 pneumonitis, other all-grade immune-related adverse events (endocrine, cutaneous, musculoskeletal, and gastrointestinal), and the number of patients that discontinuied durvalumab due to pneumonitis. Quality assessment was conducted using the method postulated by (Hoy et al., 2012). Every question was awarded a score of 1 or 0 for yes or no, respectively. The scores we combined to calculate the total score ranging from 0 to 10. Studies were then classified as low (>8), moderate (6–8), or with high (≤5) risk of bias. Classification was carried out by two reviewers independently. Any disagreements were resolved by consulting the third reviewer.

### Statistical analysis

Survival and toxicity data were extracted from the included studies and tabulated in a spreadsheet. For studies reporting data only as Kaplan-Meier survival curves, 1-year OS or PFS data was extracted from the figures using Enguage Digitizer software. All data were transformed by logit transformation and combined using the DerSimonian–Laird meta-analysis model. The analysis was conducted using “Open MetaAnalyst” software (Wallace et al., 2009). All meta-analyses were conducted using a random-effects model. Forest plots were generated to calculate pooled proportions with 95% confidence intervals (CI). Inter-study heterogeneity was checked using the I^2^ statistic. Due to the inherent limitation of the meta-analysis software, publication bias could not be checked.

We also carried out a “leave-one-out”, subgroup, and meta-regression analysis for the primary outcomes. In the “leave-one-out” meta-analysis, one study at a time was sequentially removed by the meta-analysis software and the pooled proportion was recalculated. We carried out a subgroup analysis based on the study population (Asian or Western) and the number of study centers (single-center or multicentric). Meta-regression was conducted based on the following continuous variables: median age, median time to durvalumab after CRT, the proportion of patients with ECOG-PS score of 0, PD-L1 <1%, and PD-L1 ≥50%. Bubble plots were generated for the meta-regression analysis.

## Results

### Baseline details

**FIGURE 1:**
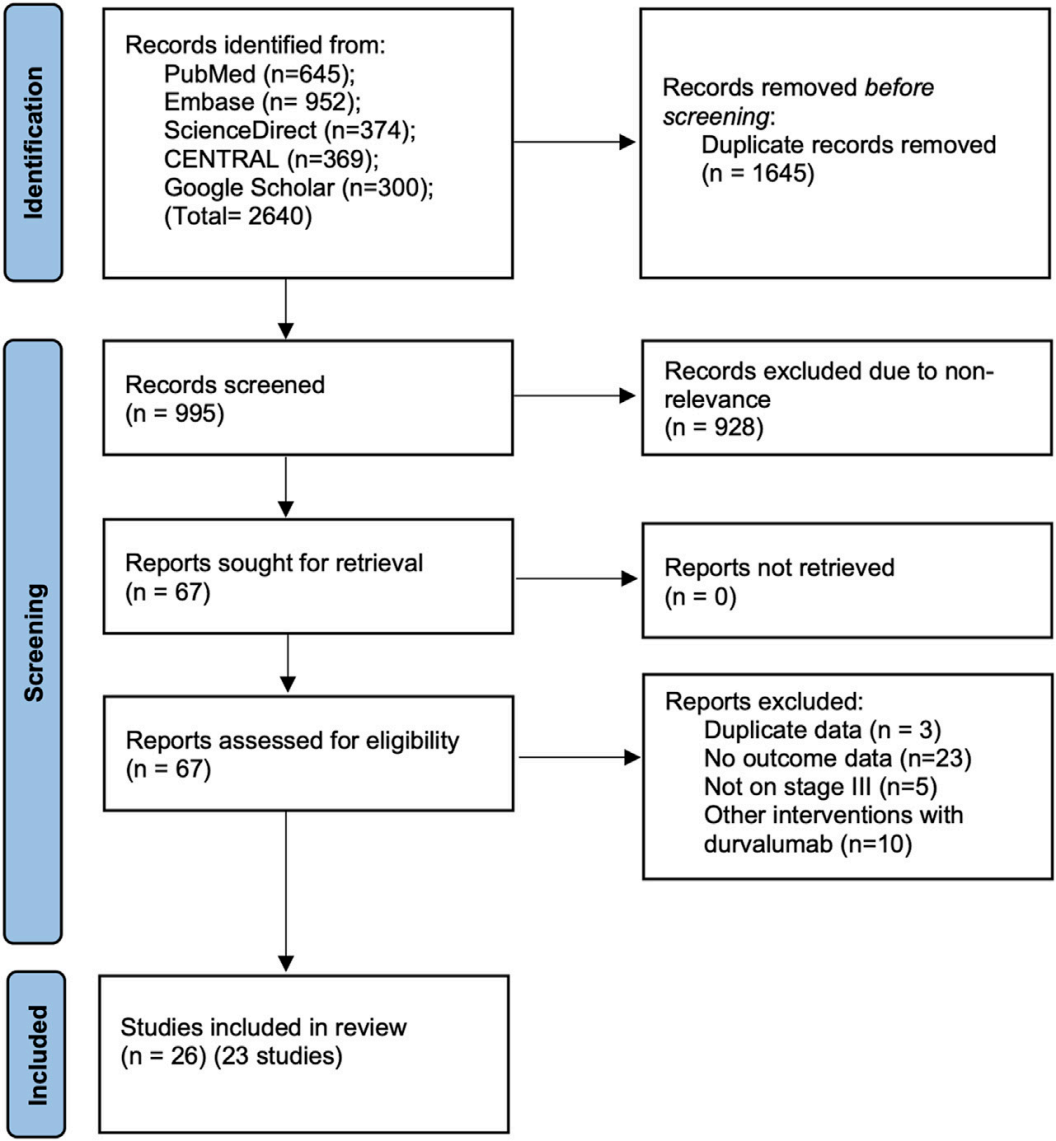
Study flow chart.

Systematic literature search across the databases identified a total of 995 unique articles (Figure 1). Of them, 67 articles were selected for a full-text review and 41 were then excluded due to different reasons. Finally, a total of 26 articles corresponding to 23 studies were included in the review (Girard et al., 2019; Girard et al., 2020; Girard et al., 2021; Faehling et al., 2020; Jain et al., 2020; Jegannathen, 2020; Jung et al., 2020; Miura et al., 2020; Noronha et al., 2020; Offin et al., 2020; Bruni et al., 2021; Desilets et al., 2021; Jazieh et al., 2021; Kartolo et al., 2021; Landman et al., 2021; Lau et al., 2021; McDonald et al., 2021; Taugner et al., 2021; Tsukita et al., 2021; Vrankar et al., 2021; Wang et al., 2021; Avrillon et al., 2022; LeClair et al., 2022; Nishimura et al., 2022; Riudavets et al., 2022; Sankar et al., 2022).

Baseline details of included studies are presented in Table 1. The studies were published between 2019 to 2022. Eleven studies were multicentric, while the remaining were single-center studies. Data were reported from several different countries around the world. A total of 4,400 patients were assessed, with the sample size of the studies ranging from 15 to 1,155 patients. The median age of patients ranged from 61 to 72 years. Eleven studies used only concomitant CRT while 8 used both cCRT and sequential CRT (sCRT). The proportion of patients with an ECOG PS score of 0 ranged from 23% to 72%. There was wide variation in the radiation dose and chemotherapy regimen used in the included studies. However, the majority of the studies used platinum-based chemotherapy. The median time from CRT to durvalumab administration ranged from 11 to 57 days. The percentage of patients with PD-L1 status <1% ranged from 9.1% to 33.3%, while those with PD-L1 ≥50% ranged from 13.4% to 46%. Quality assessment of included studies is presented in Supplementary Table S2.

**TABLE 1:** Details of included studies.

Study	Centers	Location	Sample size	Median age years (range)	ECOG-PS scrore	cCRT or sCRT	RT dose	Time from CRT to durvalumab, days (range)	Durvalumab duration (range)	Chemotherapy regimens	PD-L1 status	Driver mt	Follow-up
Desilets 2020[12]	multi	Japan & Canada	147	67	0: 36.1%	NR	NR	Median 33	NR	Platinum/vinca alkaloid 30.6%; Platinum/etoposide 21.8%;	<1%: 21.8%	NR	Median 15.8 months
					1: 57.8%			<14: 15%		Platinum/paclitaxel 25.9%;	1%–49%: 27.2%		
					2: 4.1%			14–42: 57.85		Platinum/pemetrexed 11.6%; Unknown 10.2%	≥50%: 36.1%		
								>42: 27.2%					
Faehling 2020[11]	multi	Germany	126	62.4 (34–82)	0: 48.7%	cCRT: 96.8%	Median 65 Gy	NR	Completed 1 year: 42.9%	Cisplatin 84.4%; Carboplatin 15.6%	0: 28.8%	NR	Median 25.1 months
					1: 46%	sCRT: 3.2%					1%–49%: 37.8%		
					2: 5.3%						≥50%: 33.3%		
Girard 2019–21[18,28,33,39]	multi	Multi-national^a^	1155	NR	NR	cCRT: 77.3%; sCRT: 14.1%	Median 65 Gy	Median 52 (39–89)	Median 22 infusions	NR	<1%: 11.9% ≥ 1%: 49.7%	NR	NR
Jain 2020[29]	multi	UK	28	66	NR	cCRT: 96%	66 Gy/33F: 60%	Median 39 (28–77)	Median 6 cycles (1–26)	Carboplatin/paclitaxel 53%	<1%/Unknown: 29%	NR	Median 21.3 weeks
						sCRT: 4%							
Jegannathen 2020[30]	multi	UK	18	NR	0: 72%	cCRT: 61%	66 Gy/32–33F: 61% 55 Gy/20F: 39%	NR	Median 10 infusions (2–33)	Platinum-based doublet-chemo for all	<1%: 27.8% > 1%: 72.2%	NR	1 year
					≥1: 28%	sCRT: 39%							
Jung 2020[10]	1	South Korea	21	65.9 (36–77)	0: 18%	cCRT	NR	NR	NR	NR	<1%: 23.8%	EGFR mt: 9.5%	NR
					1: 81%						1%–9%: 23.8% 10%–49%: 19% ≥ 50%: 23.8%		
Miura 2020[13]	1	Japan	41	72 (51–80)	0: 58%	cCRT	60 Gy/30F: 98%	Median 11 (1–42)	Median 6.75 doses	Carboplatin/PTX 44%; carboplatin 34%; cisplatin/docetaxel 10%; cisplatin/TS-1 8%; carboplatin/docetaxel 2%; Cisplatin/etoposide 2%	<1%: 29%	EGFR mt: 12%	Median 271 days
					1: 42%		54 Gy/25F: 2%	<14: 61%	14.6% completed 1 year treatment		1%–49%: 27% ≥ 50%: 22% unknown: 22%		
								14–42: 39%					
Noronah 2020[31]	1	India	15	61 (36–75)	NR	NR	NR	NR	NR	Cisplatin/etoposide 6.7%; Carboplatin/paclitaxel 80%; Carboplatin/pemetrexed 6.7%	<1%: 13.3%	NR	Median 9 months
											1%–50%: 60%		
											>50: 26.7%		
Offin 2020[32]	1	USA	62	66 (49–86)	0: 53%	cCRT	54–66 Gy/27–33F	Median 45 (9–231)	Median 18 doses (4–24)	NR	<1%: 34%	EGFR mt: 2%	Median 12 months
					1: 47%						1%–49%: 30% ≥ 50%: 36%		
Bruni 2021[21]	multi	Italy	155	66 (40–82)	0: 60% 1: 36.8% 2: 3.2%	cCRT: 58.8%	60 Gy/30F: 77.5%	Median 52 (9–245)	Median 13 cycles (1–34)	Platinum-based chemo for all	<1%: 9.1%	NR	Median 14 months
						sCRT: 41.2%	66 Gy/33F: 6.5% 44–54 Gy/22–27F: 6.5% 51–55 Gy/17–20F: 9.5%	<42: 22%			1%–50%: 45/8%		
								>42: 78%			>50: 40.6%		
Jazieh 2021[20]	1	USA	99	NR	NR	NR	NR	NR	NR	NR	<1%: 33.3%	EGFR mt: 2%; ALK fusion: 49%	NR
											1%–49%: 30.3%		
											≥50%: 36.4%		
Landman 2021[19]	1	Israel	39	66.5 (48.5–85.1)	0: 23% 1: 77%	cCRT	Median 69.9 Gy	Median 66 (18–159)	Median 21 cycles (1–26)	Platinum-based doublet-chemo for all	<1%: 28%	EGFR or ALK fusion: 8%	Median 20.4 months
											>1%: 46%		
											Unknown: 26%		
Lau 2021[23]	1	Canada	82	NR	NR	cCRT	NR	NR	NR	NR	NR	NR	NR
LeClair 2021[34]	1	USA	83	69.8	0: 28% 1: 65% 2: 7%	NR	Median 60 Gy	Average 57.3 (8–226)	Median 13.9 doses (1–47)	Carboplatin/paclitaxel 70%; Cisplatin/pemetrexed 12%; Cisplatin/etoposide 8%; Carboplatin/pemetrexed 5%; Carboplatin/etoposide 2%; Carboplatin/nab-paclitaxel 1%; Not mentioned 1%	<1%: 20%	NR	NR
							<60 Gy: 4% 60 Gy: 48% > 60 Gy: 16%				1%–49%: 18% ≥ 50%: 29% unknown: 33%		
Nishimura 2021[35]	1	Japan	82	69.5 (37–86)	NR	cCRT	NR	<42: 85.4%	NR	Cisplatin/S-1 29.3%;	<1%: 20.7%	NR	Median 14.5 months
										Carboplatin/paclitaxel 24.4%;	≥50%: 13.4%		
										Cisplatin/vinorelbine 22%;			
										Carboplation 13.4%;			
										Carboplatin/nab-paclitaxel 36.5%			
Taugner 2021[22]	1	Germany	26	67.6	NR	cCRT: 96%	60 Gy/30F: 92%	Median 25 (13–103)	Median 14 cycles (2–24)	Platinum-based doublet-chemo 96%	≥50%: 46%	NR	Median 20.6 months
						sCRT: 4%							
Tsukita 2021[24]	multi	Japan	107	70 (43–86)	0: 66.4%	cCRT	Median 60 Gy	NR	Median 14 doses (1–26)	Cisplatin/vinorelbine 44.9%; Carboplatin/paclitaxel 38.3%; Cisplatin/pemetrexed 9.3% Others 7.5%	<1%: 20.6%	EGFR mt: 10.3%	Median 14.3 months
					1: 33.6%						1%–49%: 27.1% ≥ 50%: 29%	ALK fusion: 3.7%	
Vrankar 2021[25]	1	Slovenia	85	63 (36–73)	0: 43.5% 1: 54.1% 2: 2.4%	cCRT: 63.5%	Median 60 Gy	Median 57 (12–99)	NR	Gemcitabine/cisplatin 92.9%;	<1%: 15.3%	NR	Median 23 months
						sCRT: 36.5%				Pemetrexed/cisplatin 3.5%	1%–49%: 38.8% ≥ 50%: 37.7%		
											Unknown: 8.2%		
Wang 2021[26]	multi	Taiwan	61	63 (32–86)	0–1: 96.7%	cCRT	50–60 Gy: 4.9%	Median 54 (6–117)	NR	Platinum-based doublet-chemo for all	<1%: 26.2%	EGFR mt: 26.2%	NR
					2: 3.3%		60–66 Gy: 86.6%				≥1%: 44/3%		
							66–70: 11.5%				Unknown: 19.7%		
Avrillon 2021[36]	multi	France	576	64 (36–85)	NR	cCRT	Median 66 Gy	Median 36 (0–157)	Median 16 doses (1–37)	Cisplatin/vinorelbine 38.2%;	<1%: 32.6%	EGFR mt: 1.8%	Median 25.1 months
										Carboplatin/paclitaxel 27.9%; Cisplatin/pemetrexed 9.8%;	≥1%: 48.1%	ALK mt: 0.5%	
										Carboplatin/vinorelbine 6.8%;	Unknown: 19.3%		
										Carboplatin/pemetrexed 6.1%;			
										Cisplatin/docetaxel 4.9%;			
										Carboplatin/etoposide 2.1%			
Kartolo 2022[27]	2	Canada	63	NR	0–1: 84%	cCRT	54–66 Gy	Mean 48 ± 117	NR	Platinum-based chemo for all	<1%: 13%	0%	Median 17 months
					≥2: 16%						1%–49%: 25% ≥ 50%: 43%		
											Unknown: 19%		
Riudavets 2022[37]	multi	Multi-national^b^	323	66 (38–85)	0–1: 98%	cCRT: 81%	NR	NR	NR	NR	<1%: 16%		Median 18.5 months
					≥2: 2%	sCRT: 19%					13.3%		

Sankar 2022[38]	multi	USA	1006	69 (64–72)	NR	cCRT	NR	Median 42 (29–63)	NR	Carboplatin/paclitaxel 70.7%;	NR	NR	Median 19.9 months
										Cisplatin/etoposide 6.16%;			
										Platinum/pemetrexed 10.5%;			
										Other 12.6%			

^a^ United Kingdom, France, Italy, Germany, Norway, Israel, Netherlands, Belgium, Australia, United States.

^b^ France, Spain, Italy, Belgium, Germany, United States, Argentina.

ALK, anaplastic lymphoma kinase; cCRT, concomitant chemoradiotherapy; ECOG-PS, eastern cooperative oncology group performance status; EGFR, epidermal growth factor receptor; F, fractions; Gy, grays; mt, mutation; NR, not reported; PD-L1, programmed death-ligand 1; RT, radiotherapy; sCRT, sequential chemoradiotherapy.

### OS

**FIGURE 2:**
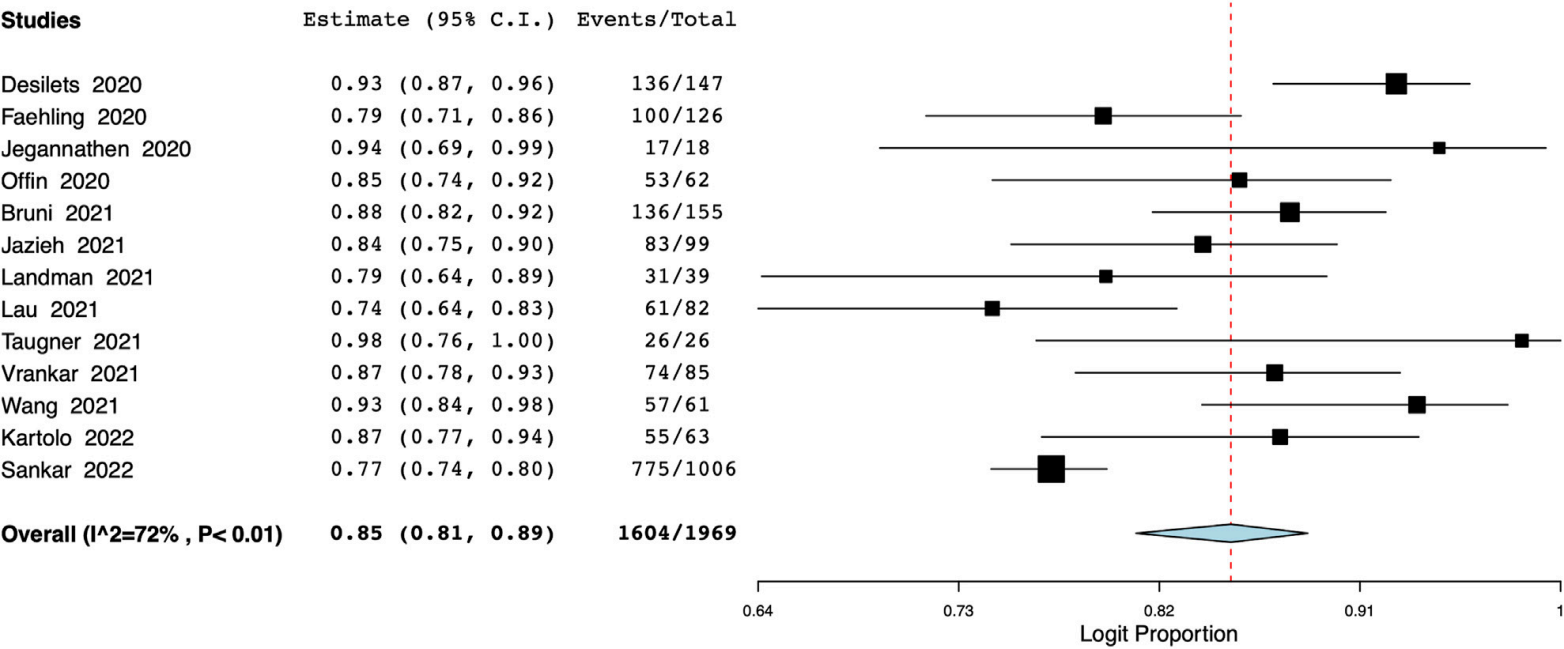
Meta-analysis of 1-year OS.

Data on 1-year OS was available from 14 studies with 1969 participants. Meta-analysis indicated a 1-year OS of 85% (95% CI: 81%–89%) (Figure 2). Inter-study heterogeneity was 72%. There was not much variation in the leave-one-out analysis with the estimate ranging from 84% to 86% (Supplementary Figure S1). On the subgroup analysis, the 1-year OS was 83% (95% CI: 79%–87%) in studies of the Western population and 88% (95% CI: 67%–96%) in the Asian population (Supplementary Figure S2). Based on the number of study centers, the OS was 83% (95% CI: 77%–87%) in single-center studies and 85% (95% CI: 79%–90%) in multicentric studies (Supplementary Figure S3). Results of the meta-regression analysis are presented in Table 2. None of the moderators were found to significantly influence the effect size. The bubble plots of the meta-regression are presented as Supplementary Figures S4–S8.

**TABLE 2:** Meta-regression analysis for the primary outcomes.

Variable	Coefficient	−95% CI	+95% CI	SE	*p*-value	Bubble plot
*1-year OS*						
Median age	−0.039	−0.182	0.105	0.073	0.599	Supplementary Figure S4
Time to durvalumab	−0.017	−0.054	0.019	0.019	0.351	Supplementary Figure S5
ECOG PS 0%	0.006	−0.021	0.034	0.014	0.657	Supplementary Figure S6
PD-L1 <1%	−0.017	−0.043	0.009	0.013	0.193	Supplementary Figure S7
PD-L1 ≥50%	0.067	−0.004	0.138	0.036	0.064	Supplementary Figure S8
*1-year PFS*						
Median age	0.028	−0.030	0.086	0.030	0.338	Supplementary Figure S12
Time to durvalumab	−0.001	−0.023	0.022	0.011	0.949	Supplementary Figure S13
ECOG PS 0%	**0.017**	**0.002**	**0.033**	**0.008**	**0.026**	Supplementary Figure S14
PD-L1 <1%	−0.009	−0.028	0.011	0.010	0.396	Supplementary Figure S15
PD-L1 ≥50%	0.312	−0.335	0.959	0.330	0.344	Supplementary Figure S16
*All-grade pneumonitis*						
Median age	**0.057**	**0.028**	**0.086**	**0.015**	**<0.001**	Supplementary Figure S20
Time to durvalumab	**−0.006**	**−0.011**	**−0.001**	**0.003**	**0.030**	Supplementary Figure S21
ECOG PS 0%	−0.001	−0.010	0.007	0.004	0.747	Supplementary Figure S22
PD-L1 <1%	−0.001	−0.016	0.014	0.008	0.895	Supplementary Figure S23
PD-L1 ≥50%	**−0.019**	**−0.033**	**−0.005**	**0.007**	**0.009**	Supplementary Figure S24

SE, standard error; CI, confidence interval; PD-L1, programmed death-ligand 1; ECOG-PS, eastern cooperative oncology group performance status; OS, overall survival; DFS, disease free survival.

Significant values highlighted bold.

### PFS

**FIGURE 3:**
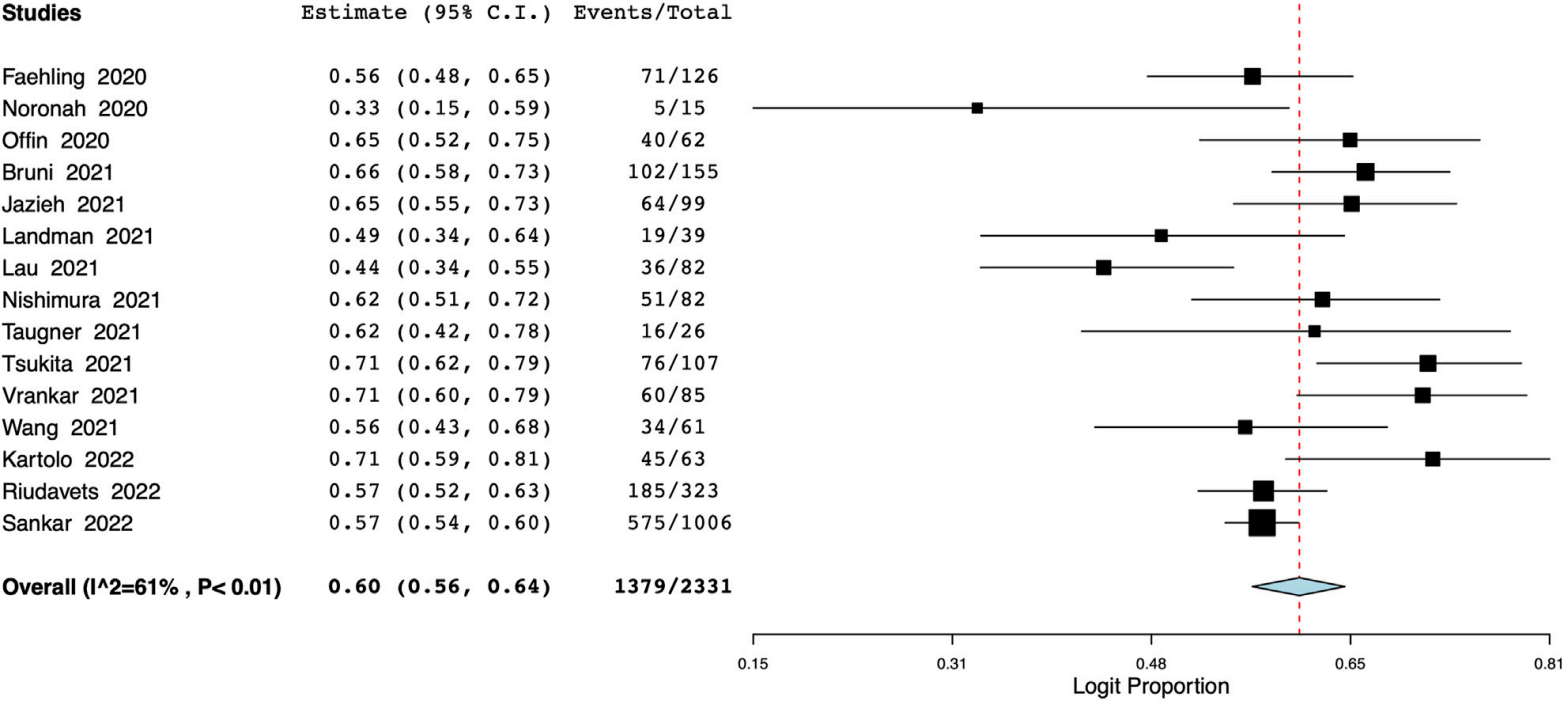
Meta-analysis of 1-year PFS.

A total of 15 studies with 2,331 patients reported data on 1-year PFS. Pooled analysis indicated a 1-year PFS of 60% (95% CI: 56%–64%) (Figure 3). Inter-study heterogeneity was 61%. On leave-one-out analysis, the pooled PFS ranged from 59% to 61% (Supplementary Figure S9). On subgroup analysis, the PFS was 61% (95% CI: 56%–65%) for studies conducted in Western countries and 58% (95% CI: 47%–68%) for Asian population studies (Supplementary Figure S10). The PFS was 58% (95% CI: 50%–66%) for single-center studies and 61% (95% CI: 57%–65%) for multicentric studies (Supplementary Figure S11). On meta-regression, median age, time to durvalumab, and proportion of patients with PD-L1 status of <1% or ≥50% did not affect the overall effect size (Table 2). However, as the proportion of patients with ECOG PS of 0% increased, and there was a corresponding statistically significant increase in the 1-year PFS (*p* = 0.026). Bubble plots of the PFS meta-regression are presented in Supplementary Figures S12–S16.

### Pneumonitis

**FIGURE 4:**
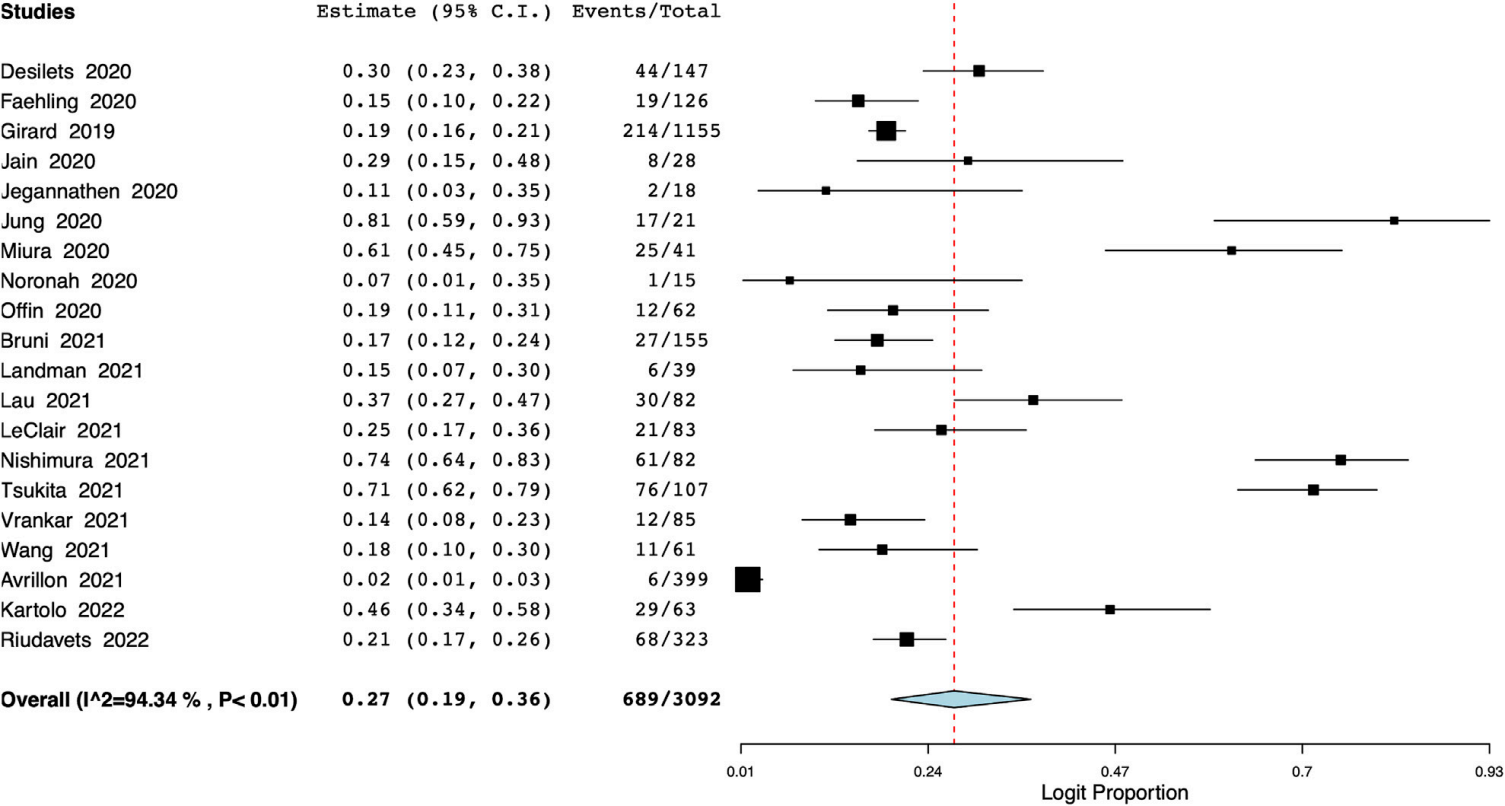
Meta-analysis of all-grade pneumonitis.

Pooled meta-analysis of 20 studies showed that the incidence of all-grade pneumonitis was 27% (95% CI: 19%–36%) (Figure 4), with the interstudy heterogeneity of 94.3%. The incidence of pneumonitis ranged from 25% to 30% on the leave-one-out meta-analysis (Supplementary Figure S17). On the subgroup analysis, the incidence was lower in Western studies [21% (95% CI: 12%–30%)] and higher in studies on Asian populations [47% (95% CI: 23%–70%)] (Supplementary Figure S18). In terms of the number of study centers, the incidence of pneumonitis was 25% (95% CI: 15%–35%) in multi-center studies and 37% (95% CI: 20%–53%) in single-center studies (Supplementary Figure S19). Meta-regression results are presented in Table 2 with bubble plots as Supplementary Figures S20–S24. On meta-regression, the incidence of all-grade pneumonitis increased significantly with increasing age (*p* < 0.001) (Table 2). Additionally, longer time between CRT and durvalumab treatment was significantly associated with a lower incidence of pneumonitis (*p* = 0.03). We also noted that as the proportion of patients with PD-L1 status of ≥50% increased, the incidence of pneumonitis decreased significantly (*p* = 0.009).

**FIGURE 5:**
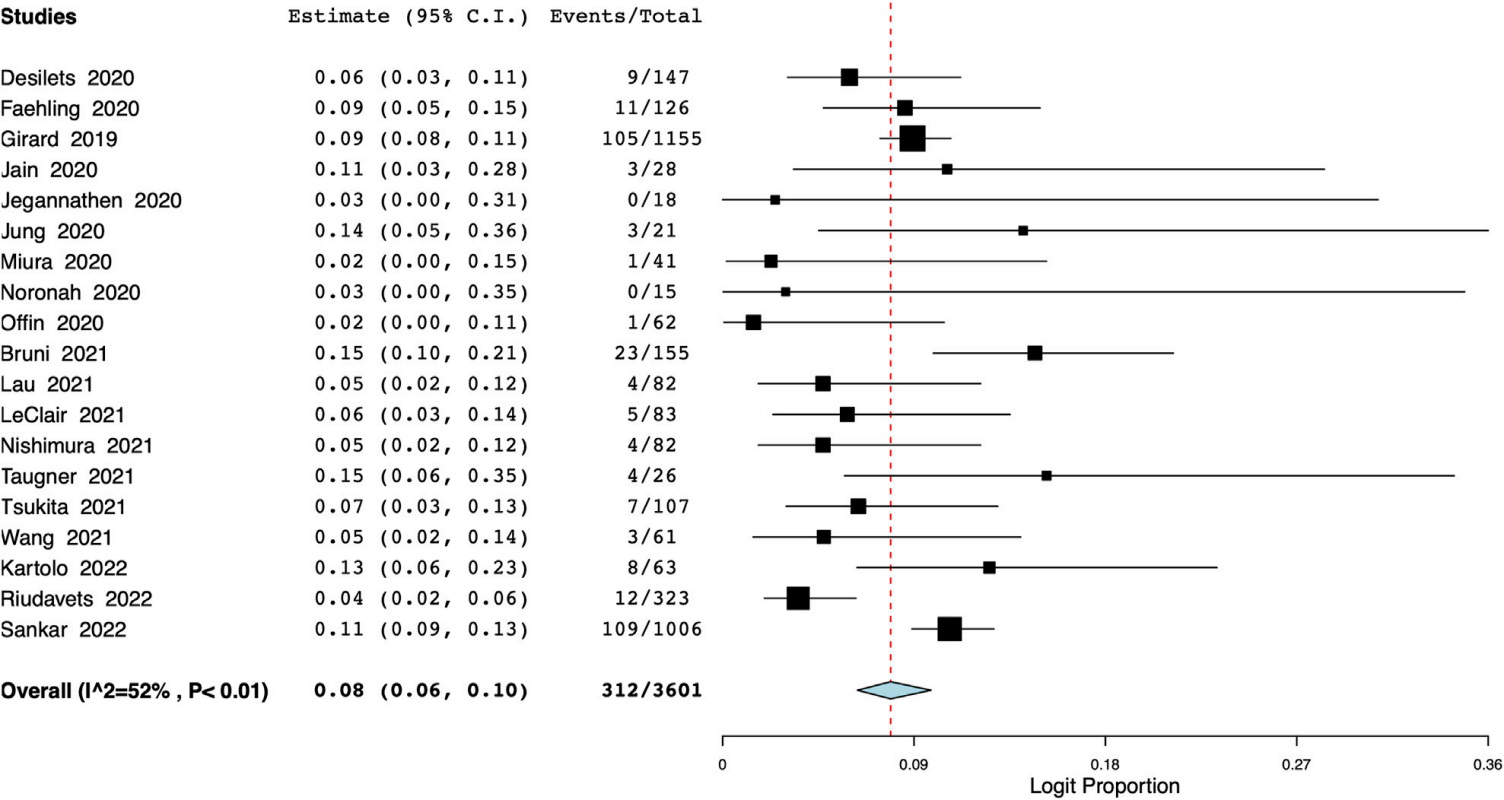
Meta-analysis of grade ≥3 pneumonitis.

**FIGURE 6:**
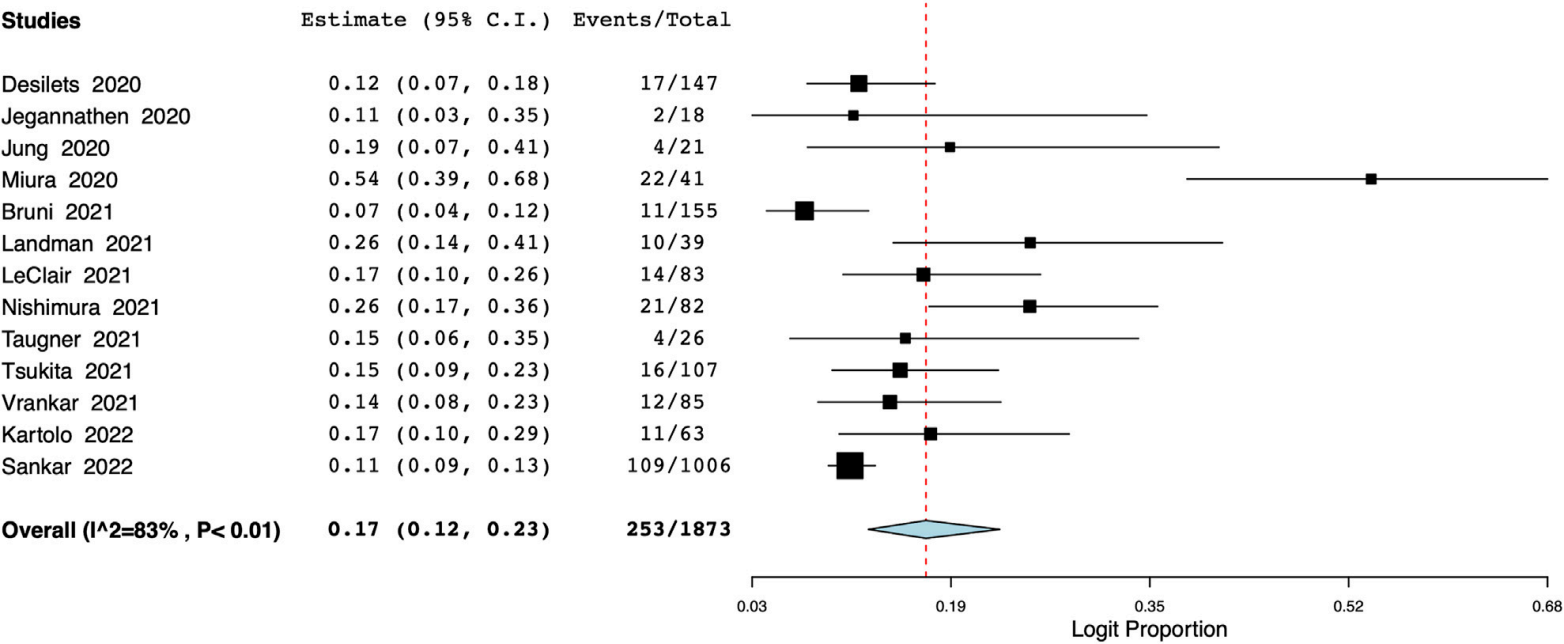
Meta-analysis of number of patients discontinuing durvalumab due to pneumonitis.

The meta-analysis also revealed that the incidence of grade ≥3 pneumonitis was 8% (95% CI: 6%–10%, I^2^ = 52%) (Figure 5). Based on data from 13 studies, the proportion of patients discontinuing durvalumab due to pneumonitis was 17% (95% CI: 12%–23%, I^2^ = 83%) (Figure 6).

### Other adverse events

**FIGURE 7:**
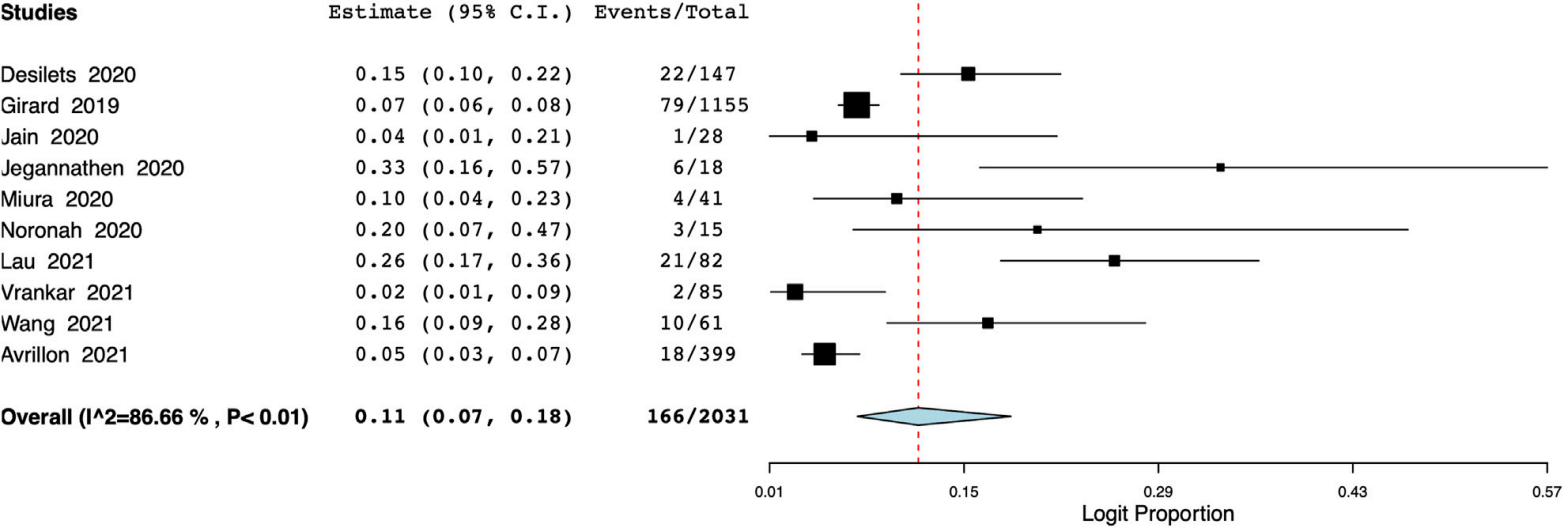
Meta-analysis of endocrine adverse events.

**FIGURE 8:**
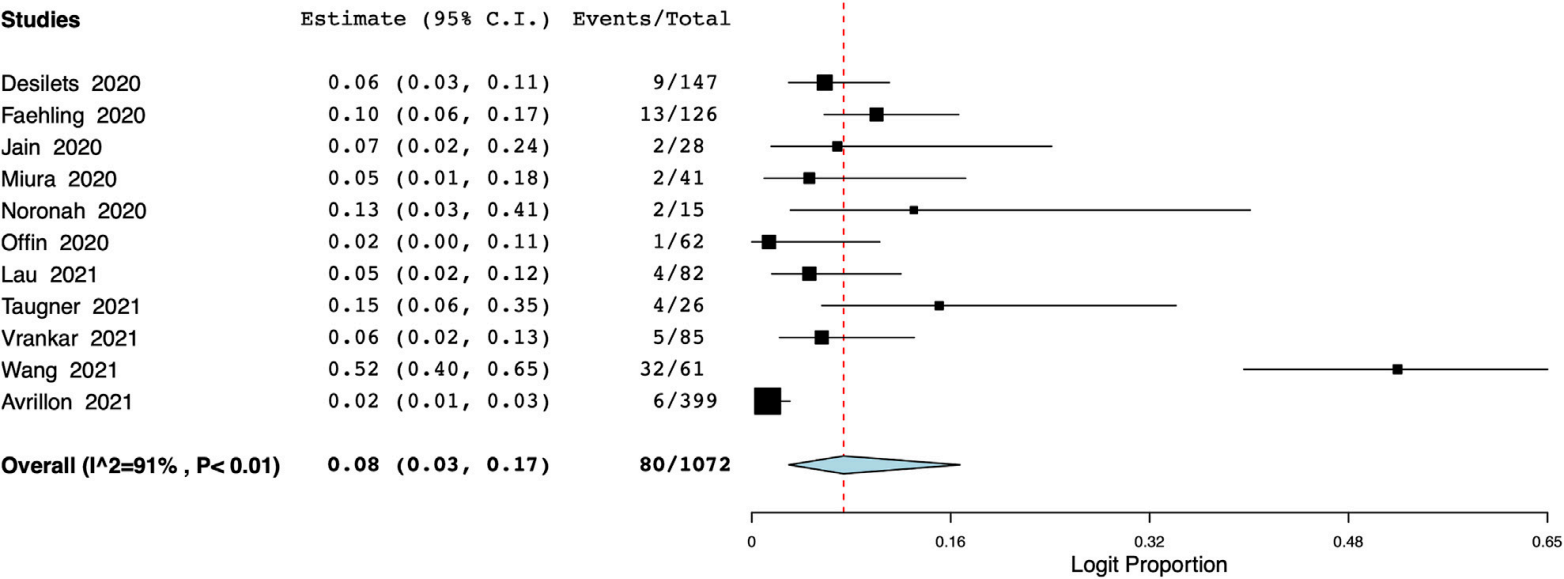
Meta-analysis of cutaneous adverse events.

**FIGURE 9:**
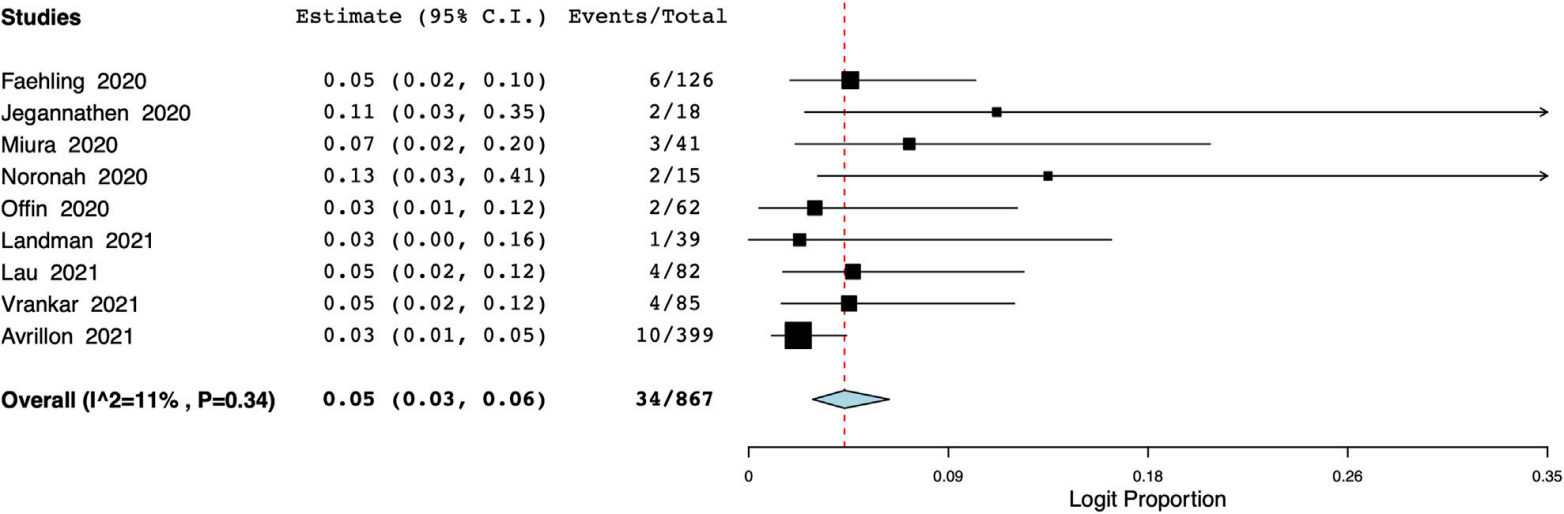
Meta-analysis of musculoskeletal adverse events.

**FIGURE 10:**
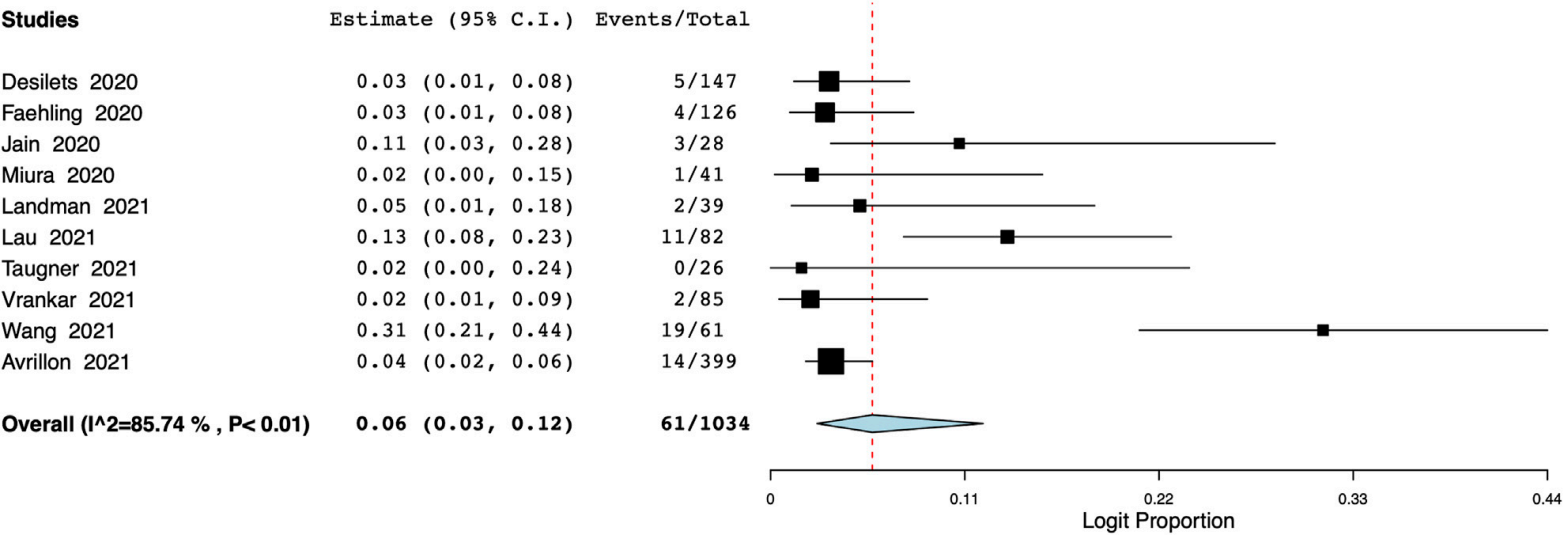
Meta-analysis of gastrointestinal adverse events.

The pooled proportion of patients experiencing endocrine adverse events was 11% (95% CI: 7%–18%, I^2^ = 86.7%) (Figure 7). Meta-analysis showed that the incidence of cutaneous adverse events was 8% (95% CI: 3%–17%, I^2^ = 91%) (Figure 8) whereas the pooled proportion of musculoskeletal and gastrointestinal adverse events was 5% (95% CI: 3%–6%, I^2^ = 11%) (Figure 9) and 6% (95% CI: 3%–12%, I^2^ = 85.7%) (Figure 10), respectively.

## Discussion

Real-world studies are an integral part of the evidence on the efficacy and safety of new anticancer drugs. They are highly valuable as complementing RCTs due to their high external validity (Blonde et al., 2018). Since the PACIFIC trial (Antonia et al., 2017), several authors have reported real-life data of treating unresectable stage III NSCLC with durvalumab after CRT. Our study presents the most updated compiled evidence on the subject.

An important advantage of real-life studies compared to their baseline RCTs is their broader inclusion criteria. This is apparent when the baseline characteristics of the patients in the PACIFIC trial (Antonia et al., 2017) and the included real-world studies are compared. Firstly, most lung cancer patients are elderly with a median age of 70 years (Bray et al., 2018). However, such patients are frequently excluded from RCTs. In the PACIFIC trial (Antonia et al., 2017), the median age of patients was 64 years while in most of the real-world included studies the median age was higher than 64. In the studies of Sankar et al. (2022), Tsukita et al. (2021) Nishimura et al. (2022), and LeClair et al. (2022), the median age the highest, at 69, 70, 69.5, and 69.8 years, respectively. This age range is much more reflective of the actual age of NSCLC patients in actual clinical practice. Secondly, in the PACIFIC trial (Antonia et al., 2017) only patients with ECOG PS of 0–1 were eligible for inclusion, which is consistent with clinical trials for other ICIs (Dall’Olio et al., 2020). Individuals with ECOG >2 represent a fragile set of patients who have a poor survival rate and are more prone to complications (Albain et al., 1991). While the US Food and Drug Administration and European Medicines Agency have approved the use of ICIs in this cohort, evidence for such patients is usually extrapolated from RCTs. It can be noted that many of the real-world studies in this review included patients with ECOG >2. Thirdly, only patients undergoing cCRT were included in the PACIFIC trial (Antonia et al., 2017). While cCRT achieves better survival as compared to sCRT, sCRT is still used in elderly cohorts and in patients with large tumor volumes (Xiao and Hong, 2021). Lastly, the time from CRT to durvalumab was set as <42 days in the PACIFIC trial (Antonia et al., 2017). This duration was exceeded in many studies, included in our review.

Despite these differences in baseline characteristics, our real-world analysis demonstrated that the 1-year OS and PFS were in line with the PACIFIC trial (Antonia et al., 2017). In fact, the OS and PFS in our analysis were only marginally higher (our review vs. PACIFIC; OS: 85% vs. 83.1% and PFS: 60% vs. 55.9%) (Antonia et al., 2017; Antonia et al., 2018). In comparison, the previous real-world review of Wang et al. (2022), which included only five studies in the survival analysis, reported a 1-year OS (379 patients) and PFS (330 patients) of 90% and 62%, respectively. In the current review, the OS and PFS analysis included 14 and 15 studies with 1969 and 1,379 patients, respectively, potentially providing better estimate of the real-world data of the actual survival rates. The higher survival rates in real-world studies could be due to the heterogeneity in the use of Response Evaluation Criteria for Solid Tumors amongst different centers leading to overestimation of treatment efficacy (Wang et al., 2022). Secondly, patient follow-up and screening may not be as rigorous in clinical practice as compared to clinical trials which too could raise the survival figures.

The results of our meta-regression analysis showed that an increased proportion of patients with an ECOG score of 0 had better PFS. ECOG represents the ability of patients to care for themselves and a physical ability in terms of walking, working, or time spent confined to a bed or chair during waking hours (Dall’Olio et al., 2020). A lower score indicates a healthier patient which may have influenced the PFS rates. However, we noted that median age did not influence OS and PFS. Treatment of older cancer patients is challenging as the clinician needs to balance between toxicities of combined multiple treatments and a potential curative effect. Furthermore, due to poor ECOG status and multiple comorbidities such patients are frequently undertreated (Socinski et al., 2021). In line with our results, Lau et al. (2021) have reported that elderly patients receiving consolidation durvalumab after CRT for stage III NSCLC have similar overall response rates, OS, and PFS as compared to their younger counterparts. Post-hoc analysis of the PACIFIC trial also suggests similar efficacy of durvalumab in elderly patients (Socinski et al., 2021). However, these results must be interpreted with caution due to the selection bias in real-world studies and the small sample size of elderly patients in RCTs.

We reported that median time from CRT to durvalumab did not influence the survival rates. Unlike the PACIFIC trial (Antonia et al., 2017) that only included patients receiving durvalumab within 42 days, real-world studies included patients beyond this time range. Indeed, patients in the PACIFIC trial (Antonia et al., 2017) were healthier (as demonstrated by their age and ECOG status) and could recover quickly from the adverse events of CRT. The prolonged time of durvalumab initiation in the real world points out to the heterogeneous population requiring longer time to recover from the toxicities of CRT. Additionally, logistic delays may occur in the real-world setting due to off-protocol systemic treatment planning and administration. In the PACIFIC trial (Antonia et al., 2018), OS was reduced in patients initiating durvalumab after 14 days. However, Desilets et al. (2021) have found no impact of durvalumab timing on OS in a real-world setting. Further studies are needed to assess the association of the time lag between CRT and durvalumab with the survival rates.

The association between PD-L1 expression and survival with ICI is unclear. Some studies suggest that higher PD-L1 expression results in better treatment outcomes with ICIs (Herbst et al., 2014; Rizvi et al., 2015), while others suggest otherwise (Brody et al., 2017). Due to heterogeneity of reporting in the included studies, we used two moderators for the meta-regression analysis, namely, the proportion of patients with PD-L1<1% and ≥50%. However, we noted no relationship between PD-L1 expression and survival. Similarly, the PACIFIC trial investigators noted no difference in OS and PFS in different subgroups based on PD-L1 status, although the trial was not initially designed to interpret such difference (Antonia et al., 2018). Similar results were reported by the real-world studies of Offin et al. (2020), Desilets et al. (2021) and Faehling et al. (2020). However, Jazieh et al. (2021) demonstrated that patients with >50% PD-L1 expression had significantly longer PFS and OS than those with lower PD-L1 expression. This variability in the results may be due to the variability in testing for PD-L1 status and the sample size. Future RCTs stratifying patients based on PD-L1 expression are needed for stronger evidence.

The rates of endocrinal, cutaneous, and musculoskeletal toxicities in our analysis were 11%, 8%, and 5%, respectively, while in the PACIFIC trial (Antonia et al., 2017) they were 11%, 7%, and 5%, respectively. The incidence of all-grade pneumonitis was slightly lower in our analysis as compared to the PACIFIC trial (Antonia et al., 2017) (27% vs. 33.9%). However, we reported higher incidence of grade ≥3 pneumonitis (8% vs. 4%) and more frequent discontinuation of therapy due to pneumonitis (17% vs. 4.8%) compared to the PACIFIC trial data. A higher incidence of severe pneumonitis and more frequent rate of therapy discontinuation in real-world studies could be due to baseline differences in the study population. Sankar et al. (2022) in their retrospective analysis of the US veterans cohort noted a higher proportion of smokers and baseline lung disease in the veteran’s cohort as compared to the PACIFIC trial (Antonia et al., 2017). Both these factors are associated with severe disease (Nishino et al., 2016). We noted higher rates of pneumonitis in Asian population as compared to Western studies, suggesting a role of ethnicity in the incidence of pneumonitis. Furthermore, studies with a higher proportion of elderly patients had increased pneumonitis rates. Similar results were reported by the previous review (Wang et al., 2022). We showed that delays in durvalumab administration reduced the incidence of pneumonitis. It is known that radiation pneumonitis is associated with thoracic radiotherapy. While significant advances have been made to limit the incidence of radiation pneumonitis using 3D conformational techniques and intensity-modulated radiotherapy, the use of radiotherapy combined with immunotherapy is still linked to higher incidence of pneumonitis. In most cases, it is difficult to distinguish between radiation and immune-mediated pneumonitis (LeClair et al., 2022). Thus, a delay in durvalumab could have decreased the burden of radiation pneumonitis in the studies by allowing recovery time and reducing the overall incidence of pneumonitis. Lastly, our analysis indicated a reduced incidence of pneumonitis with an increased proportion of patients with PD-L1 expression of ≥50%. However, previous studies showed that high PD-1 expression correlates with an increased risk of pneumonitis. Increased PD-L1 leads to increased T-cell activation, which can cause collateral damage to normal lung tissue manifesting as pneumonitis (Chao et al., 2022). Such contradictive results are difficult to explain since we noted no such relationship when PD-L1 expression <1% was used as a moderator for the same analysis. More research is needed to assess the role of PD-L1 expression and the risk of pneumonitis associated with durvalumab treatment.

Our review has some limitations. First, real-world studies are observational and are prone to bias due to errors in record-keeping or data entry. The role of selection bias in influencing results cannot be excluded. Secondly, there was high interstudy heterogeneity in most of our results, which was expected due to variability in study populations, patient characteristics, and treatment protocols. We attempted to explore the source of such heterogeneity by subgroup and meta-regression analyses. Thirdly, many of the included studies had a small number of patients with limited follow-up. All outcome data were not universally reported, which reduced the number of studies in each meta-analysis. Fourthly, our analysis consisted of only single-arm studies. Future real-world studies comparing outcomes of unresectable stage III NSCLC with and without durvalumab can provide optimal complementary evidence to the PACIFIC trial (Antonia et al., 2017). Lastly, since follow-up of most included studies was short, we could assess only 1-year survival rates.

Nevertheless, our review presents the most comprehensive and up-to-date real-world evidence on the efficacy of durvalumab after CRT for unresectable stage III NSCLC. The pooled figures generated by our review may be used as a guide to physicians treating the heterogeneous cohort of stage III NSCLC patients in clinical practice that are usually not eligible or healthy enough for participation in clinical trials. These figures also provide a real-world estimation of the efficacy and safety of the drug and would help in patient-doctor interactions. The congruence of the results of our review and the PACIFIC trial (Antonia et al., 2017) further supports the use of durvalumab in improving outcomes in unresectable stage III NSCLC.

## Conclusion

Real-world evidence suggests that the short-term efficacy and safety of durvalumab are consistent with that of the PACIFIC trial. A higher proportion of severe pneumonitis is seen in clinical practice leading to treatment discontinuation. There is a need for further research analyzing the impact of various confounders like age, ethnicity, time to durvalumab, and PD-L1 status on the overall outcomes.

## Data Availability

Publicly available datasets were analyzed in this study. This data can be found here: The data that support the findings of this study are openly available in (PROSPERO) at (https://www.crd.york.ac.uk/prospero/display_record.php?ID=CRD42022324663), reference number (No CRD42022324663).

## Supplementary material

SUPPLEMENTARY FIGURE S1 Leave-one-out meta-analysis for 1-year OS.

SUPPLEMENTARY FIGURE S2 Subgroup analysis based on location for 1-year OS.

SUPPLEMENTARY FIGURE S3 Subgroup analysis based on study centers for 1-year OS.

SUPPLEMENTARY FIGURE S4 Meta-regression plot of 1-year OS for the moderator median age.

SUPPLEMENTARY FIGURE S5 Meta-regression plot of 1-year OS for the moderator time to durvalumab.

SUPPLEMENTARY FIGURE S6 Meta-regression plot of 1-year OS for the moderator ECOG PS 0%.

SUPPLEMENTARY FIGURE S7 Meta-regression plot of 1-year OS for the moderator PD L1 <1%.

SUPPLEMENTARY FIGURE S8 Meta-regression plot of 1-year OS for the moderator PD L1 ≥50%.

SUPPLEMENTARY FIGURE S9 Leave-one-out meta-analysis for 1-year PFS.

SUPPLEMENTARY FIGURE S10 Subgroup analysis based on location for 1-year PFS.

SUPPLEMENTARY FIGURE S11 Subgroup analysis based on study centers for 1-year PFS.

SUPPLEMENTARY FIGURE S12 Meta-regression plot of 1-year PFS for the moderator median age.

SUPPLEMENTARY FIGURE S13 Meta-regression plot of 1-year PFS for the moderator time to durvalumab.

SUPPLEMENTARY FIGURE S14 Meta-regression plot of 1-year PFS for the moderator ECOG PS 0%.

SUPPLEMENTARY FIGURE S15 Meta-regression plot of 1-year PFS for the moderator PD L1 <1%.

SUPPLEMENTARY FIGURE S16 Meta-regression plot of 1-year PFS for the moderator PD L1 ≥50%.

SUPPLEMENTARY FIGURE S17 Leave-one-out meta-analysis for all grade pneumonitis.

SUPPLEMENTARY FIGURE S18 Subgroup analysis based on location for 1-year PFS.

SUPPLEMENTARY FIGURE S19 Subgroup analysis based on study centers for 1-year PFS.

SUPPLEMENTARY FIGURE S20 Meta-regression plot of 1-year PFS for the moderator median age.

SUPPLEMENTARY FIGURE S21 Meta-regression plot of 1-year PFS for the moderator time to durvalumab.

SUPPLEMENTARY FIGURE S22 Meta-regression plot of 1-year PFS for the moderator ECOG PS 0%.

SUPPLEMENTARY FIGURE S23 Meta-regression plot of 1-year PFS for the moderator PD L1 <1%.

SUPPLEMENTARY FIGURE S24 Meta-regression plot of 1-year PFS for the moderator PD L1 ≥50%.
