# Supplementary material for: Efficacy and safety of consolidation durvalumab after chemoradiation therapy for stage III non-small-cell lung cancer: a systematic review, meta-analysis, and meta-regression of real-world studies
Source: Front Pharmacol. 2023 Jun 8;14:1103927. doi: 10.3389/fphar.2023.1103927 (PMC10285075; doi:10.3389/fphar.2023.1103927)
Supplement: Supplementary file 1 [file Table1.DOCX]

Supplementary table S2: Risk of bias analysis for included studies.

| **Study** | Was the study’s target population a close representation of the region’s population? | Was the sampling frame a true or close representation of the target population? | Was some form of random  selection used to select the sample, OR, was a census undertaken? | Was the likelihood of non-response bias minimal? | Were data collected  directly from the subjects (as opposed to a proxy)? | Was an acceptable case definition used in the study? | Was the study instrument that measured the parameter of interest shown to have reliability and validity (if necessary)? | Was the same mode of data collection used for all subjects? | Was the length of the shortest prevalence period for the parameter of interest appropriate? | Were the numerator(s) and denominator(s) for the parameter of interest appropriate? | Risk of study bias: low (>8), moderate (6–8) or high (≤5) |
| --- | --- | --- | --- | --- | --- | --- | --- | --- | --- | --- | --- |
| Desilets 2020[12] | No | No | No | Yes | Yes | Yes | Yes | Yes | Yes | Yes | Moderate |
| Faehling 2020[11] | Yes | Yes | No | Yes | Yes | Yes | Yes | Yes | Yes | Yes | Low |
| Girard 2019-21[18,28,33,39] | Yes | Yes | No | Yes | Yes | Yes | Yes | Yes | Yes | Yes | Low |
| Jain 2020[29] | No | No | No | Yes | Yes | Yes | Yes | Yes | No | Yes | Moderate |
| Jegannathen 2020[30] | No | No | No | Yes | Yes | Yes | Yes | Yes | Yes | Yes | Moderate |
| Jung 2020[10] | No | No | No | Yes | Yes | Yes | Yes | Yes | Yes | Yes | Moderate |
| Miura 2020[13] | No | No | No | Yes | Yes | Yes | Yes | Yes | Yes | Yes | Moderate |
| Noronah 2020[31] | No | No | No | Yes | Yes | Yes | Yes | Yes | No | Yes | Moderate |
| Offin 2020[32] | No | No | No | Yes | Yes | Yes | Yes | Yes | Yes | Yes | Moderate |
| Bruni 2021[21] | Yes | Yes | No | Yes | Yes | Yes | Yes | Yes | Yes | Yes | Low |
| Jazieh 2021[20] | No | No | No | Yes | Yes | Yes | Yes | Yes | Yes | Yes | Moderate |
| Landman 2021[19] | No | No | No | Yes | Yes | Yes | Yes | Yes | Yes | Yes | Moderate |
| Lau 2021[23] | No | No | No | Yes | Yes | Yes | Yes | Yes | Yes | Yes | Moderate |
| LeClair 2021[34] | No | No | No | Yes | Yes | Yes | Yes | Yes | No | Yes | Moderate |
| Nishimura 2021[35] | No | No | No | Yes | Yes | Yes | Yes | Yes | Yes | Yes | Moderate |
| Taugner 2021[22] | No | No | No | Yes | Yes | Yes | Yes | Yes | Yes | Yes | Moderate |
| Tsukita 2021[24] | Yes | Yes | No | Yes | Yes | Yes | Yes | Yes | Yes | Yes | Low |
| Vrankar 2021[25] | Yes | Yes | No | Yes | Yes | Yes | Yes | Yes | Yes | Yes | Low |
| Wang 2021[26] | No | No | No | Yes | Yes | Yes | Yes | Yes | Yes | Yes | Moderate |
| Avrillon 2021[36] | Yes | Yes | No | Yes | Yes | Yes | Yes | Yes | Yes | Yes | Low |
| Kartolo 2022[27] | No | No | No | Yes | Yes | Yes | Yes | Yes | Yes | Yes | Moderate |
| Riudavets 2022[37] | Yes | Yes | No | Yes | Yes | Yes | Yes | Yes | Yes | Yes | Low |
| Sankar 2022[38] | Yes | Yes | No | Yes | Yes | Yes | Yes | Yes | Yes | Yes | Low |
