# Supplementary material for: Efficacy and safety of consolidation durvalumab after chemoradiation therapy for stage III non-small-cell lung cancer: a systematic review, meta-analysis, and meta-regression of real-world studies
Source: Front Pharmacol. 2023 Jun 8;14:1103927. doi: 10.3389/fphar.2023.1103927 (PMC10285075; doi:10.3389/fphar.2023.1103927)
Supplement: Supplementary file 2 [file DataSheet1.PDF]

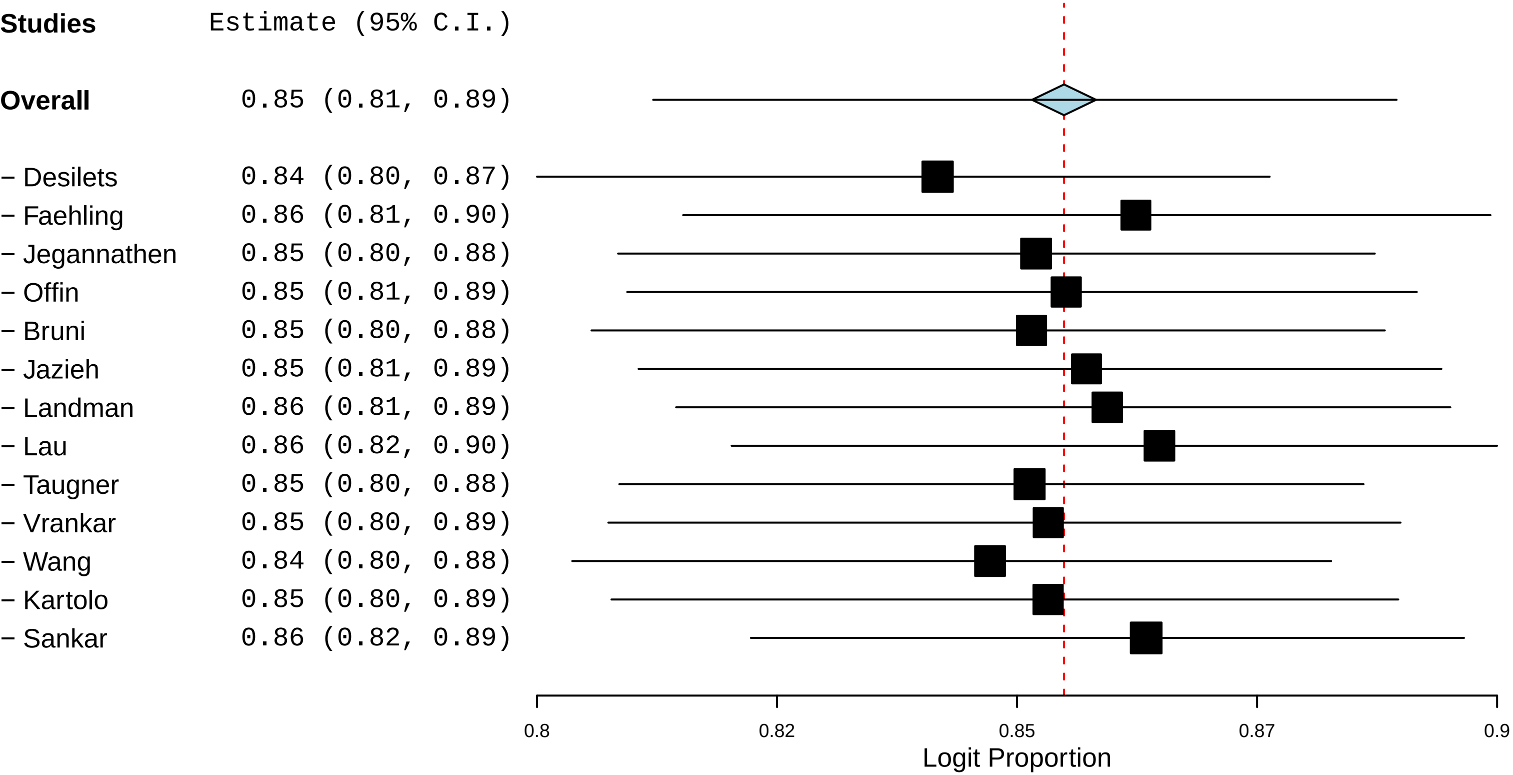
Studies
Estimate (95% C.I.)
Overall 0.85 (0.81, 0.89)
– Desilets 0.84 (0.80, 0.87)
– Faehling 0.86 (0.81, 0.90)
– Jegannathen 0.85 (0.80, 0.88)
– Offin 0.85 (0.81, 0.89)
– Bruni 0.85 (0.80, 0.88)
– Jazieh 0.85 (0.81, 0.89)
– Landman 0.86 (0.81, 0.89)
– Lau 0.86 (0.82, 0.90)
– Taugner 0.85 (0.80, 0.88)
– Vrankar 0.85 (0.80, 0.89)
– Wang 0.84 (0.80, 0.88)
– Kartolo 0.85 (0.80, 0.89)
– Sankar 0.86 (0.82, 0.89)
0.8
0.82
0.85
0.87
0.9
Logit Proportion

Studies	Estimate (95% C.I.)	Events/Total
Faehling	0.79 (0.71, 0.86)	100/126
Jegannathen	0.94 (0.69, 0.99)	17/18
Offin	0.85 (0.74, 0.92)	53/62
Bruni	0.88 (0.82, 0.92)	136/155
Jazieh	0.84 (0.75, 0.90)	83/99
Lau	0.74 (0.64, 0.83)	61/82
Taugner	0.98 (0.76, 1.00)	26/26
Vrankar	0.87 (0.78, 0.93)	74/85
Kartolo	0.87 (0.77, 0.94)	55/63
Sankar	0.77 (0.74, 0.80)	775/1006
**Subgroup Western (I^2=63% , P=0.00)**	**0.83 (0.79, 0.87)**	**1380/1722**
Landman	0.79 (0.64, 0.89)	31/39
Wang	0.93 (0.84, 0.98)	57/61
**Subgroup Asia (I^2=75% , P=0.05)**	**0.88 (0.67, 0.96)**	**88/100**
**Overall (I^2=64% , P=0.00)**	**0.84 (0.80, 0.87)**	**1468/1822**

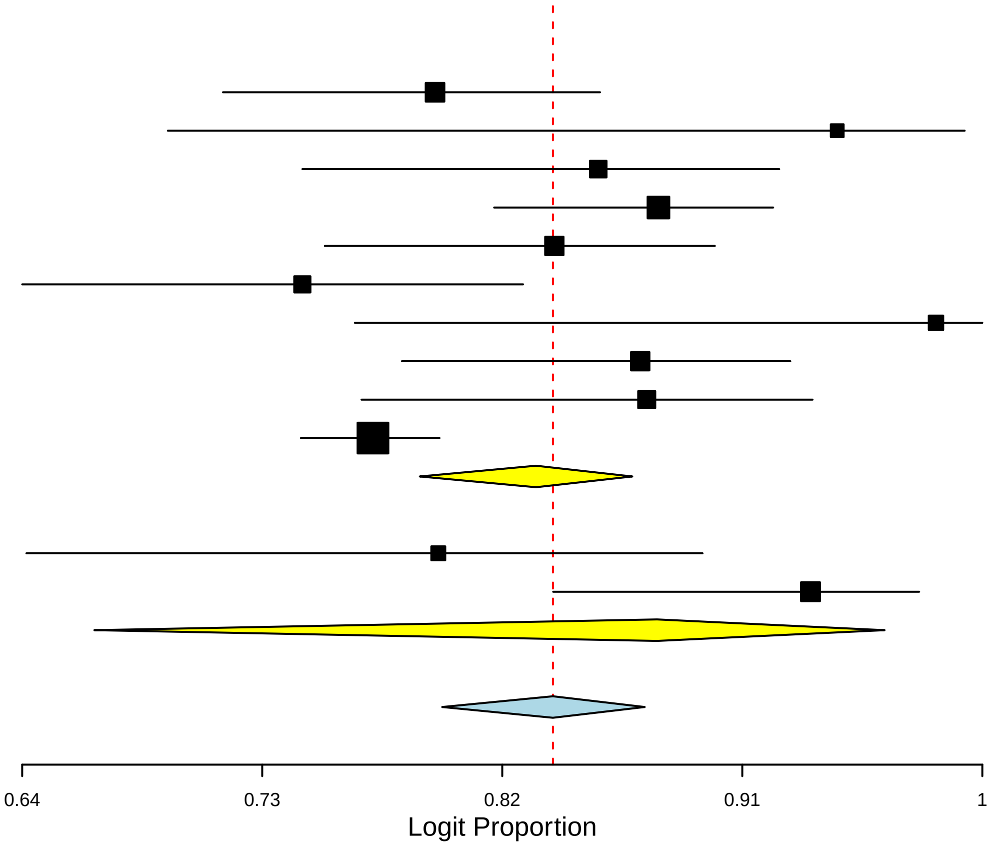

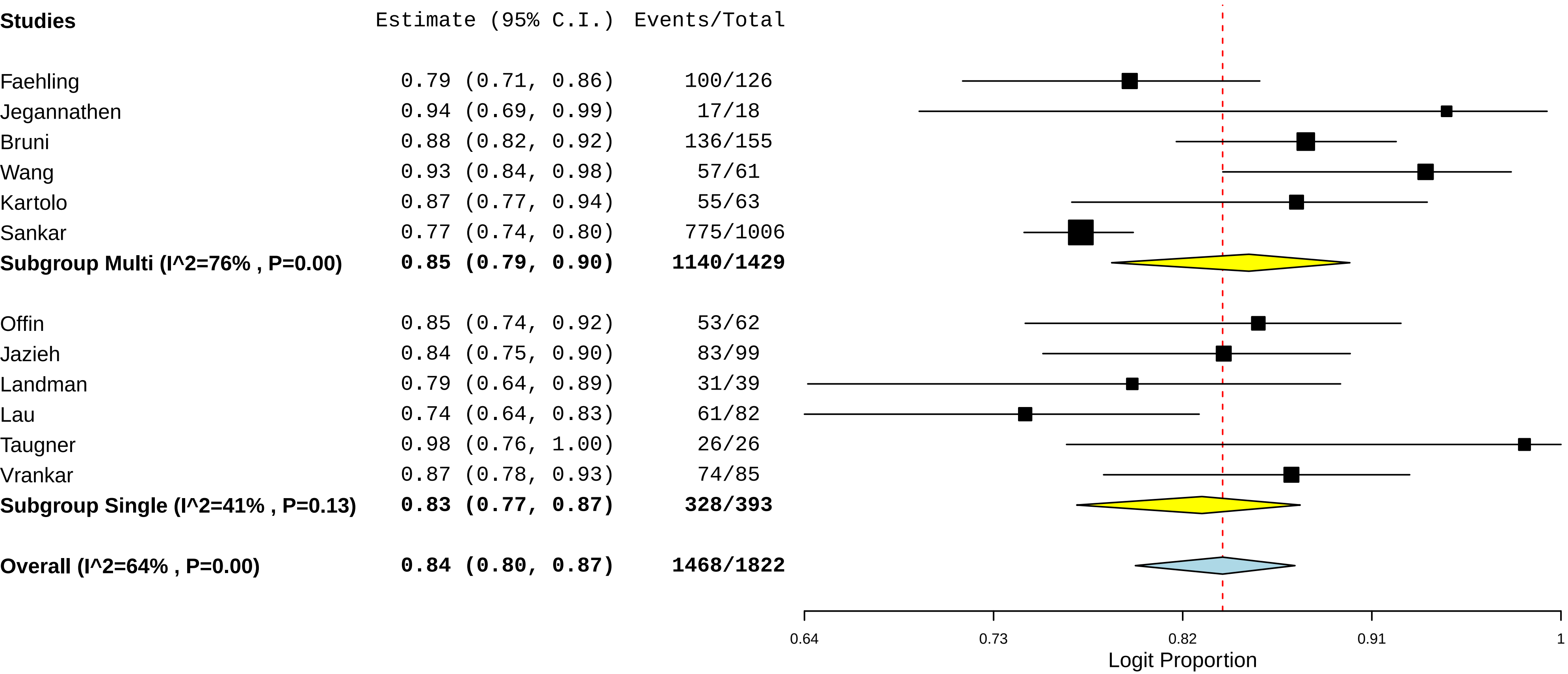
Studies
Estimate (95% C.I.)
Events/Total
Faehling 0.79 (0.71, 0.86) 100/126
Jegannathen 0.94 (0.69, 0.99) 17/18
Bruni 0.88 (0.82, 0.92) 136/155
Wang 0.93 (0.84, 0.98) 57/61
Kartolo 0.87 (0.77, 0.94) 55/63
Sankar 0.77 (0.74, 0.80) 775/1006
Subgroup Multi (I^2=76% , P=0.00) 0.85 (0.79, 0.90) 1140/1429
Offin 0.85 (0.74, 0.92) 53/62
Jazieh 0.84 (0.75, 0.90) 83/99
Landman 0.79 (0.64, 0.89) 31/39
Lau 0.74 (0.64, 0.83) 61/82
Taugner 0.98 (0.76, 1.00) 26/26
Vrankar 0.87 (0.78, 0.93) 74/85
Subgroup Single (I^2=41% , P=0.13) 0.83 (0.77, 0.87) 328/393
Overall (I^2=64% , P=0.00) 0.84 (0.80, 0.87) 1468/1822
0.64
0.73
0.82
0.91
1
Logit Proportion

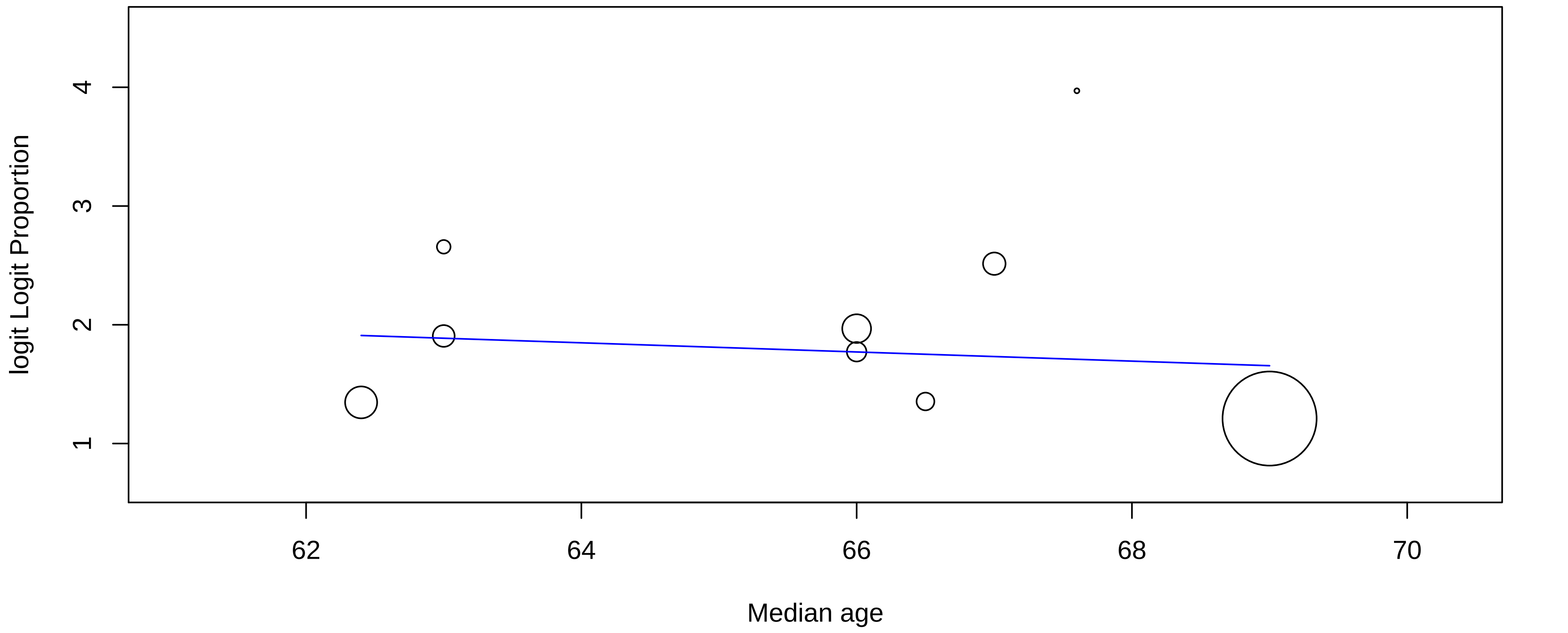

logit Logit Proportion
1
2
3
4
62
64
66
68
70
Median age

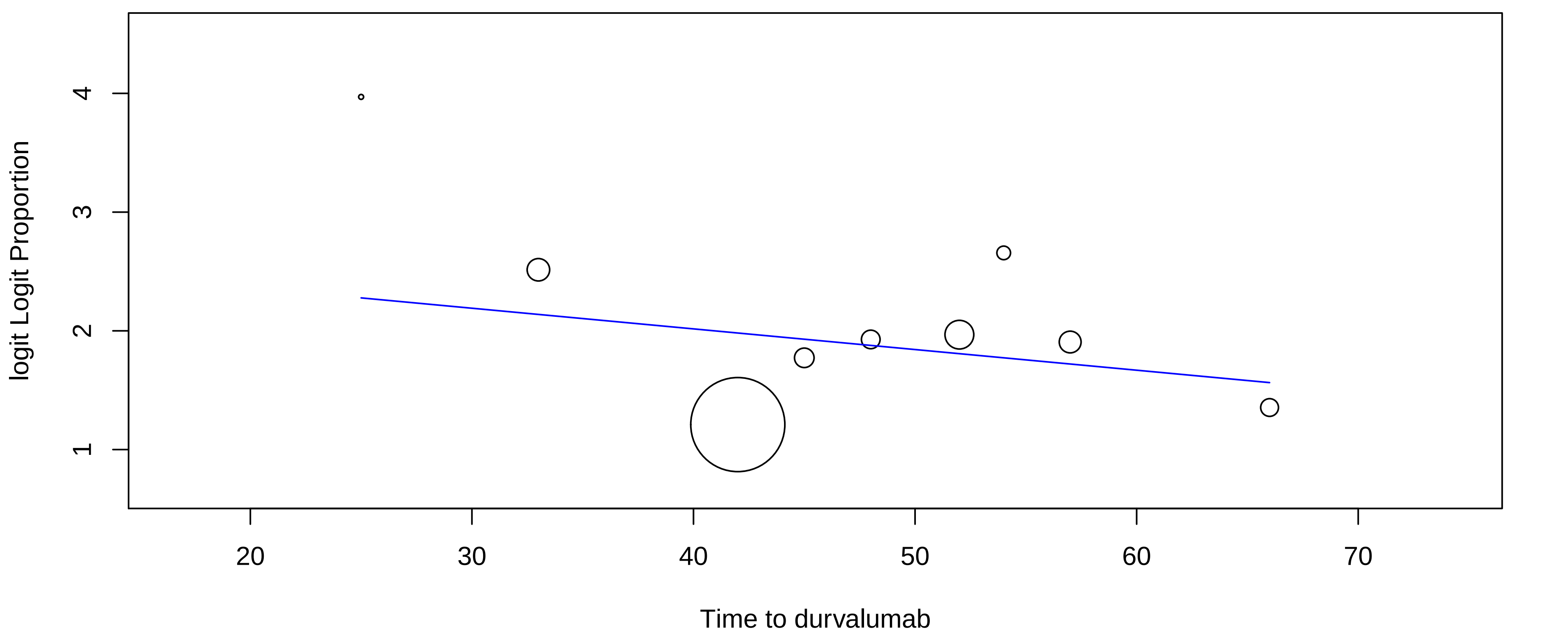

logit Logit Proportion
1
2
3
4
20
30
40
50
60
70
Time to durvalumab

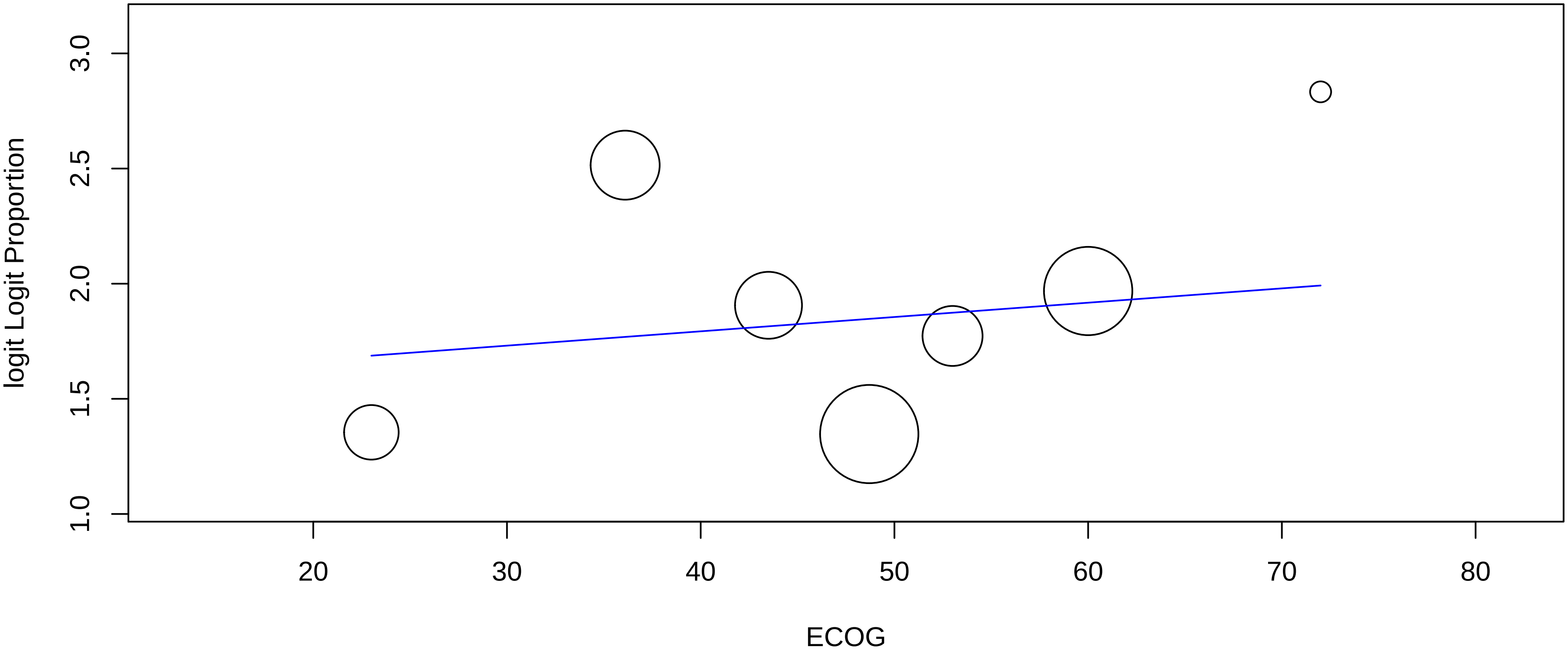
logit Logit Proportion
1.0
1.5
2.0
2.5
3.0
20
30
40
50
60
70
80
ECOG

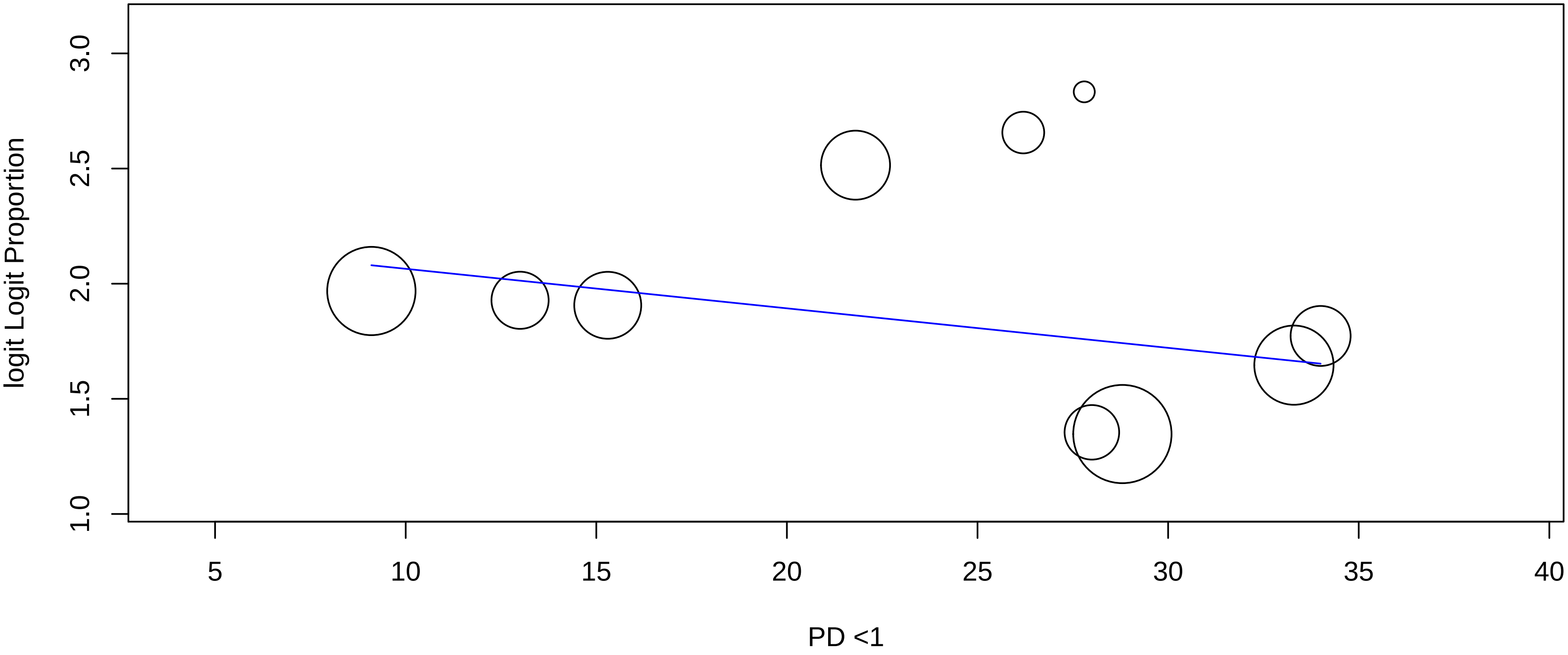

logit Logit Proportion
1.0
1.5
2.0
2.5
3.0
5
10
15
20
25
30
35
40
PD <1

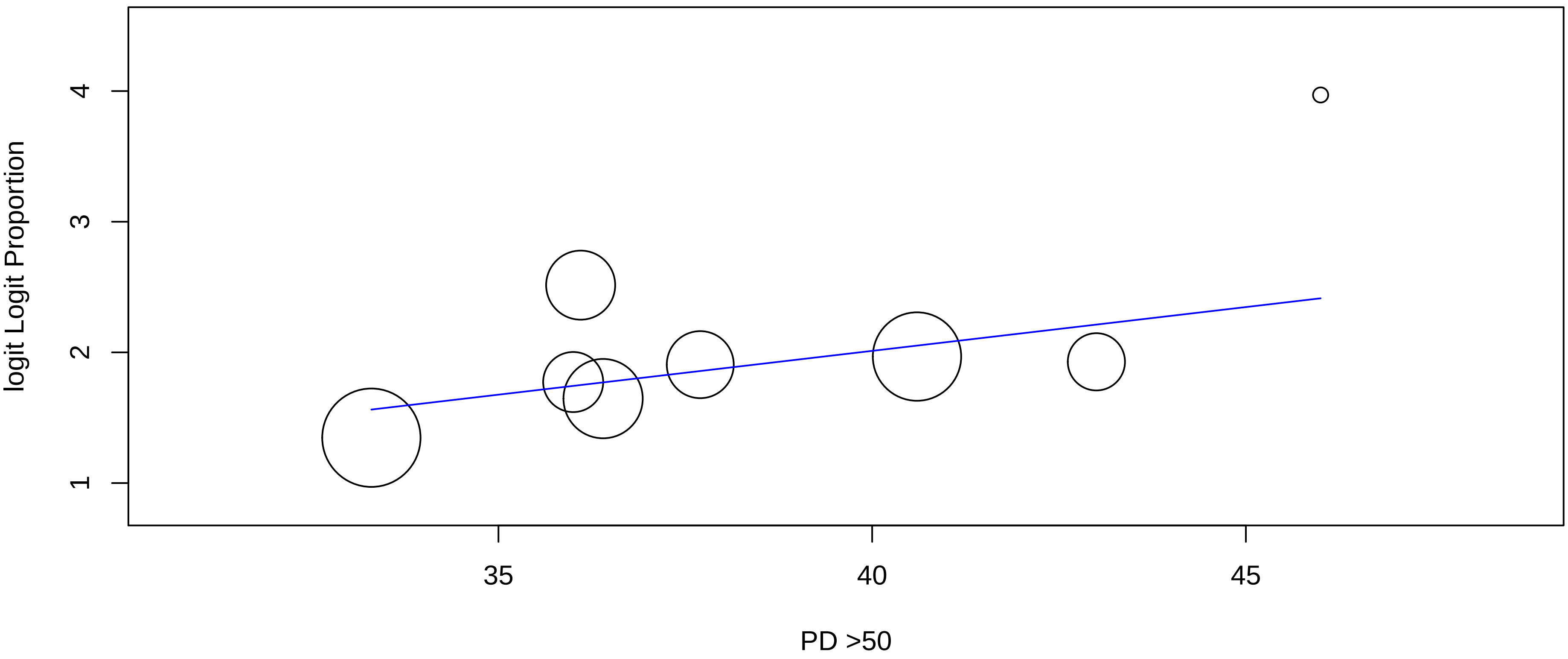

logit Logit Proportion
1
2
3
4
35
40
45
PD >50

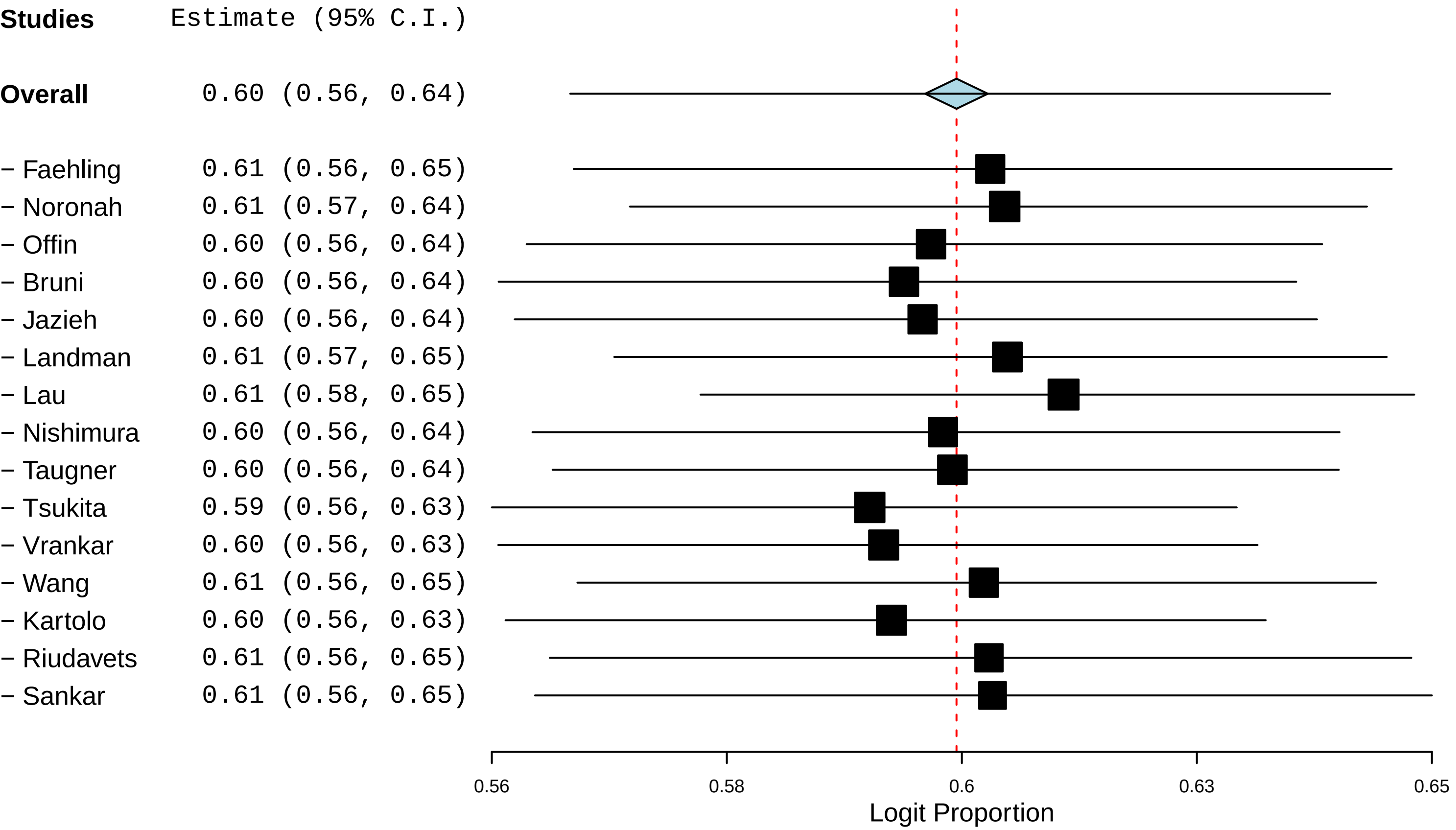

Studies
Estimate (95% C.I.)
Overall
0.60 (0.56, 0.64)
− Faehling
0.61 (0.56, 0.65)
− Noronah
0.61 (0.57, 0.64)
− Offin
0.60 (0.56, 0.64)
− Bruni
0.60 (0.56, 0.64)
− Jazieh
0.60 (0.56, 0.64)
− Landman
0.61 (0.57, 0.65)
− Lau
0.61 (0.58, 0.65)
− Nishimura
0.60 (0.56, 0.64)
− Taugner
0.60 (0.56, 0.64)
− Tsukita
0.59 (0.56, 0.63)
− Vrankar
0.60 (0.56, 0.63)
− Wang
0.61 (0.56, 0.65)
− Kartolo
0.60 (0.56, 0.63)
− Riudavets
0.61 (0.56, 0.65)
− Sankar
0.61 (0.56, 0.65)
0.56
0.58
0.6
0.63
0.65
Logit Proportion

Studies	Estimate (95% C.I.)	Events/Total
Faehling	0.56 (0.48, 0.65)	71/126
Offin	0.65 (0.52, 0.75)	40/62
Bruni	0.66 (0.58, 0.73)	102/155
Jazieh	0.65 (0.55, 0.73)	64/99
Lau	0.44 (0.34, 0.55)	36/82
Taugner	0.62 (0.42, 0.78)	16/26
Vrankar	0.71 (0.60, 0.79)	60/85
Kartolo	0.71 (0.59, 0.81)	45/63
Riudavets	0.57 (0.52, 0.63)	185/323
Sankar	0.57 (0.54, 0.60)	575/1006
**Subgroup Western (I^2=61% , P=0.01)**	**0.61 (0.56, 0.65)**	**1194/2027**
Noronah	0.33 (0.15, 0.59)	5/15
Landman	0.49 (0.34, 0.64)	19/39
Nishimura	0.62 (0.51, 0.72)	51/82
Tsukita	0.71 (0.62, 0.79)	76/107
Wang	0.56 (0.43, 0.68)	34/61
**Subgroup Asia (I^2=67% , P=0.02)**	**0.58 (0.47, 0.68)**	**185/304**
**Overall (I^2=61% , P=0.00)**	**0.60 (0.56, 0.64)**	**1379/2331**

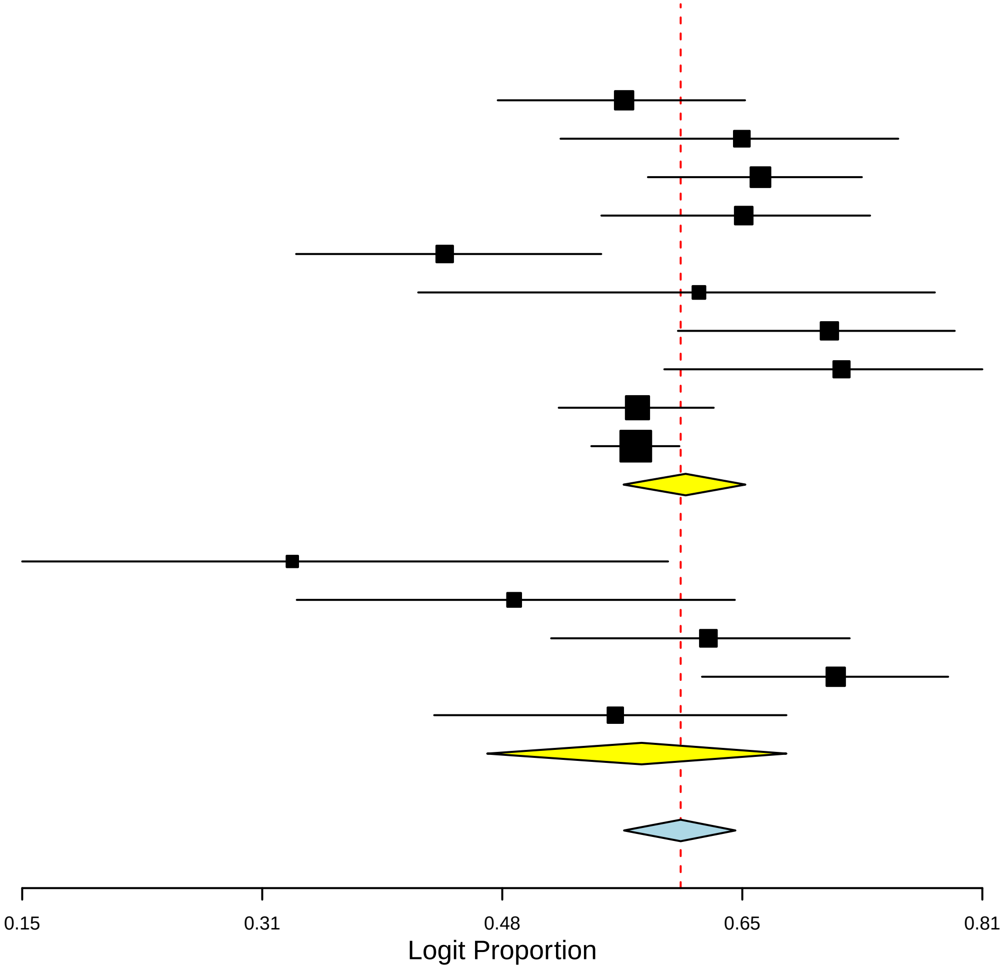

**Studies**	Estimate (95% C.I.)	Events/Total
Faehling	0.56 (0.48, 0.65)	71/126
Bruni	0.66 (0.58, 0.73)	102/155
Tsukita	0.71 (0.62, 0.79)	76/107
Wang	0.56 (0.43, 0.68)	34/61
Kartolo	0.71 (0.59, 0.81)	45/63
Riudavets	0.57 (0.52, 0.63)	185/323
Sankar	0.57 (0.54, 0.60)	575/1006
**Subgroup Multi (I^2=61% , P=0.02)**	**0.61 (0.57, 0.65)**	**1088/1841**
Noronah	0.33 (0.15, 0.59)	5/15
Offin	0.65 (0.52, 0.75)	40/62
Jazieh	0.65 (0.55, 0.73)	64/99
Landman	0.49 (0.34, 0.64)	19/39
Lau	0.44 (0.34, 0.55)	36/82
Nishimura	0.62 (0.51, 0.72)	51/82
Taugner	0.62 (0.42, 0.78)	16/26
Vrankar	0.71 (0.60, 0.79)	60/85
**Subgroup Single (I^2=65% , P=0.01)**	**0.58 (0.50, 0.66)**	**291/490**
**Overall (I^2=61% , P=0.00)**	**0.60 (0.56, 0.64)**	**1379/2331**

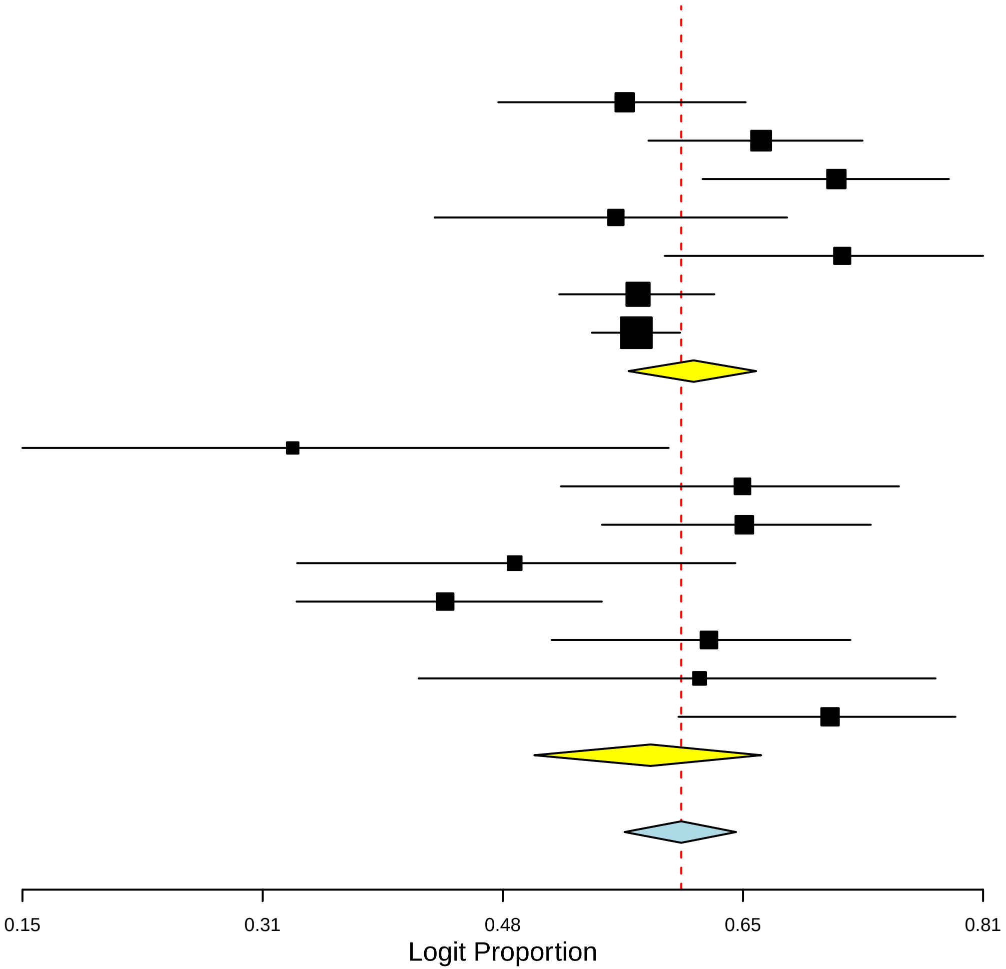

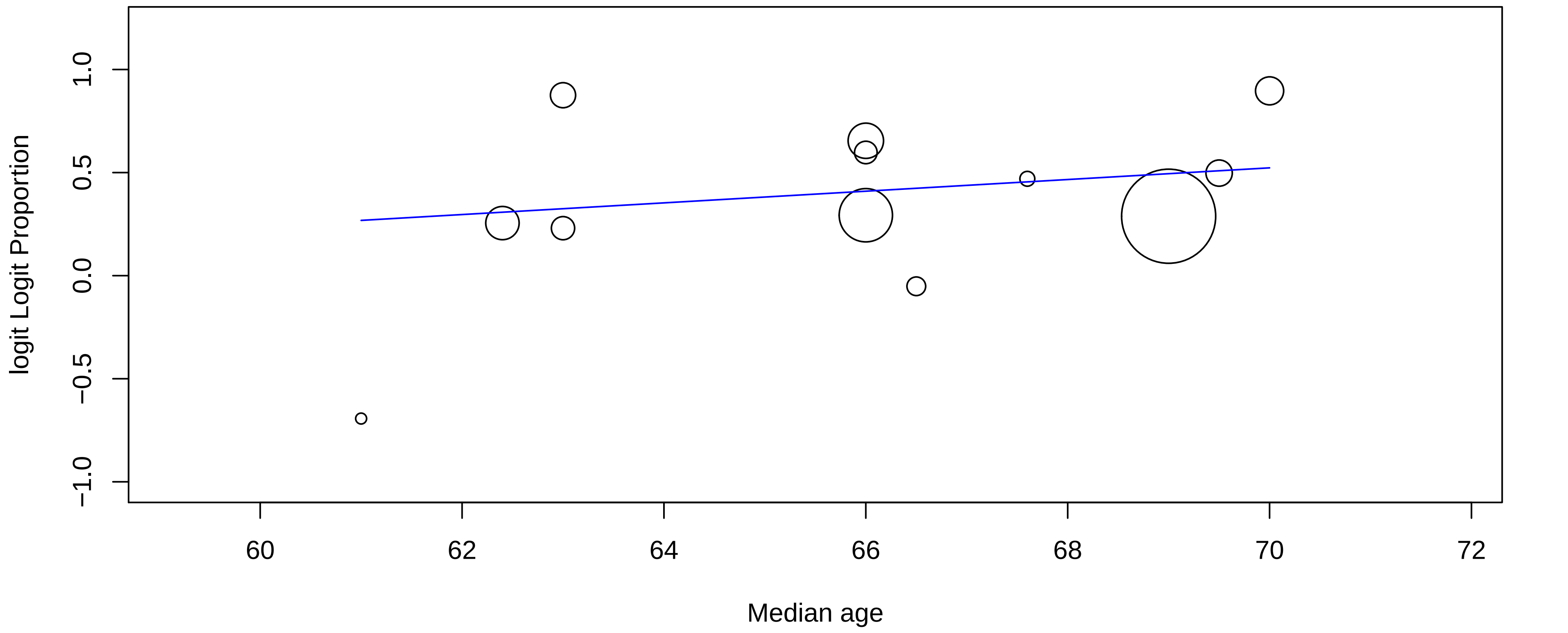
logit Logit Proportion
1.0
0.5
0.0
−0.5
−1.0
60
62
64
66
68
70
72
Median age

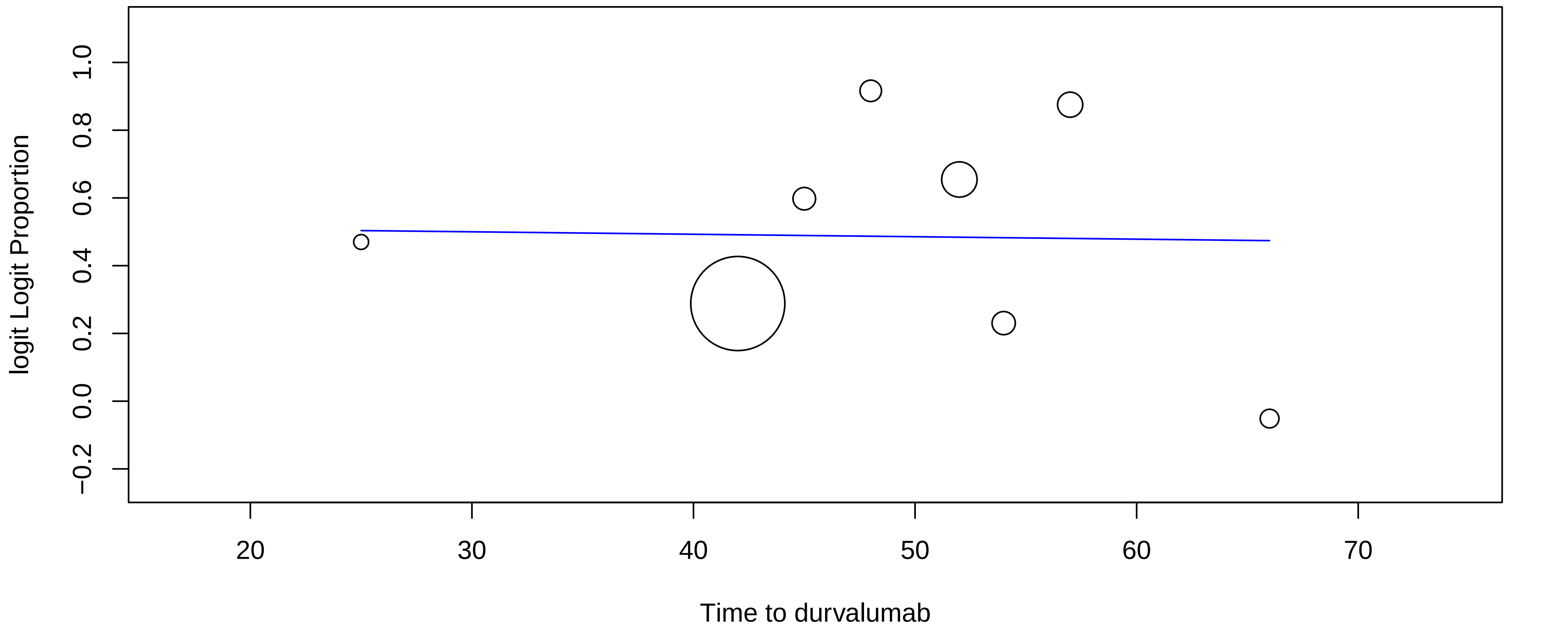

logit Logit Proportion
−0.2
0.0
0.2
0.4
0.6
0.8
1.0
20
30
40
50
60
70
Time to durvalumab

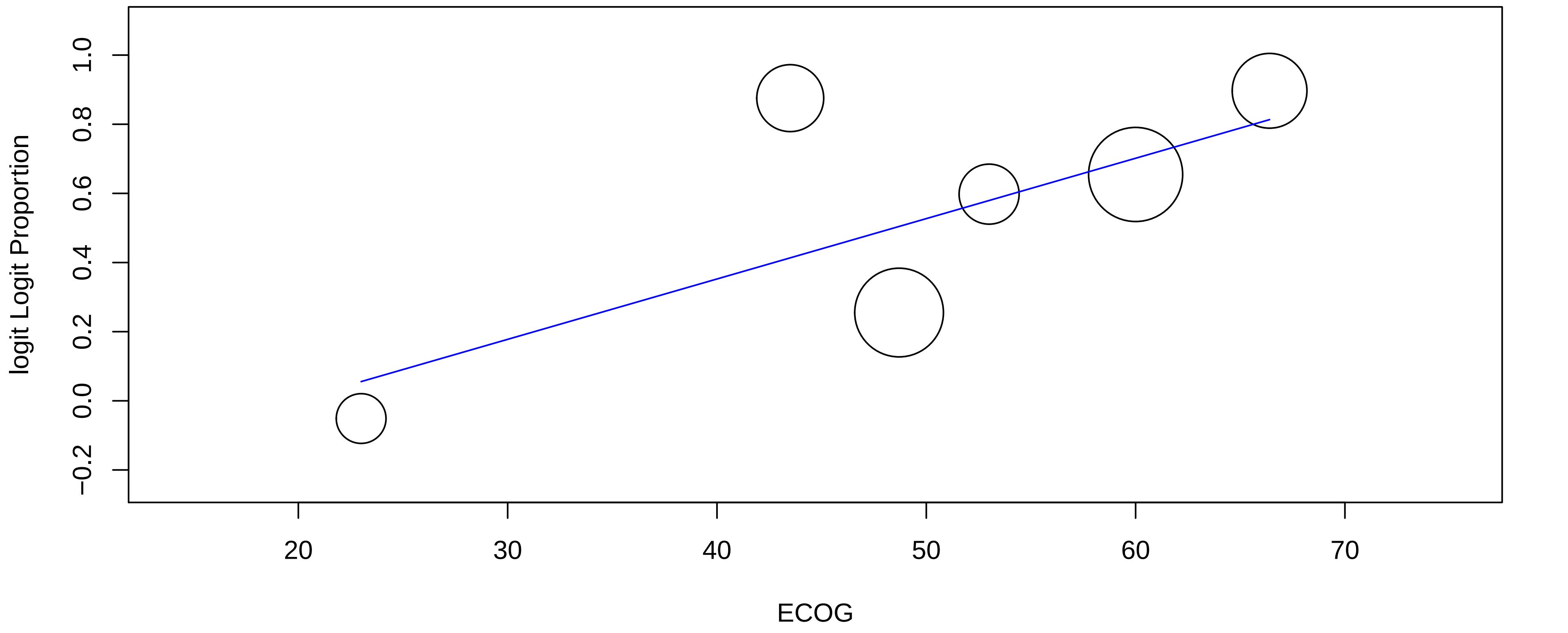

logit Logit Proportion
−0.2
0.0
0.2
0.4
0.6
0.8
1.0
20
30
40
50
60
70
ECOG

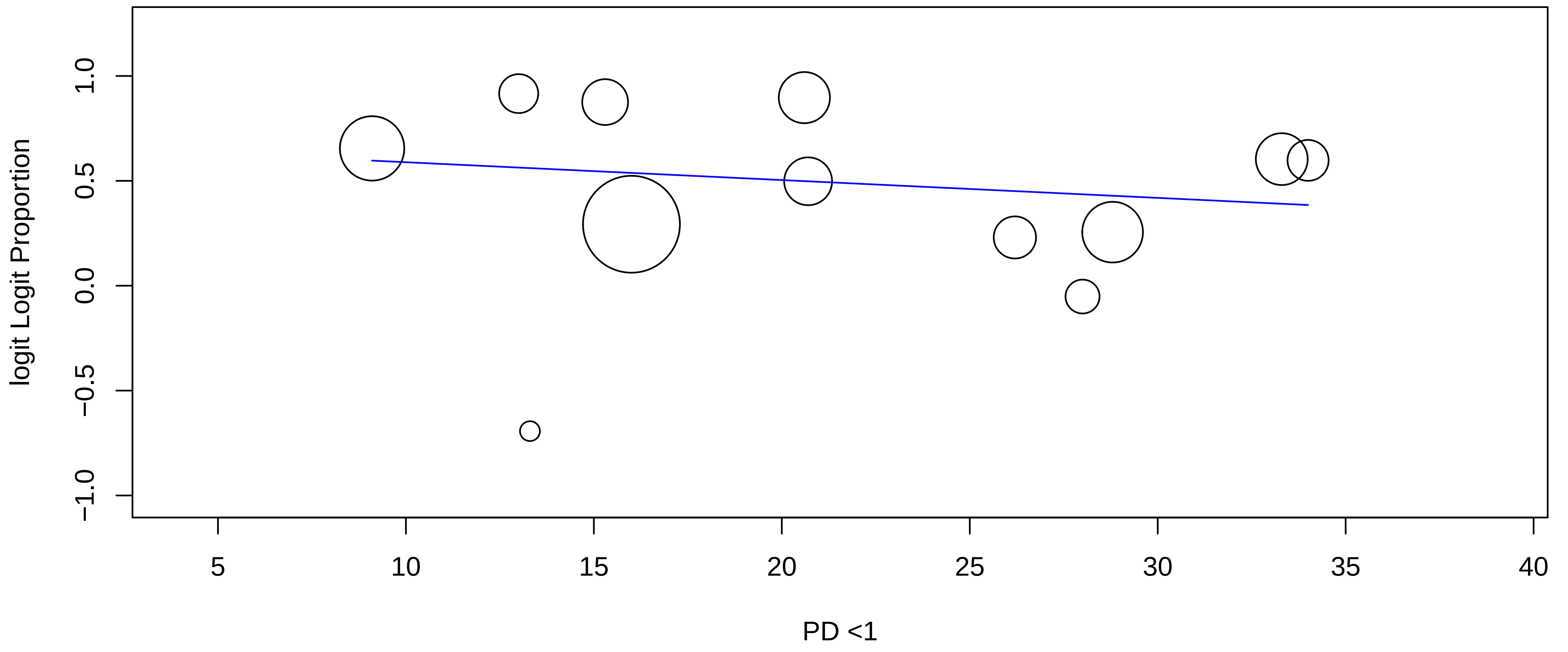

logit Logit Proportion
1.0
0.5
0.0
−0.5
−1.0
5
10
15
20
25
30
35
40
PD <1

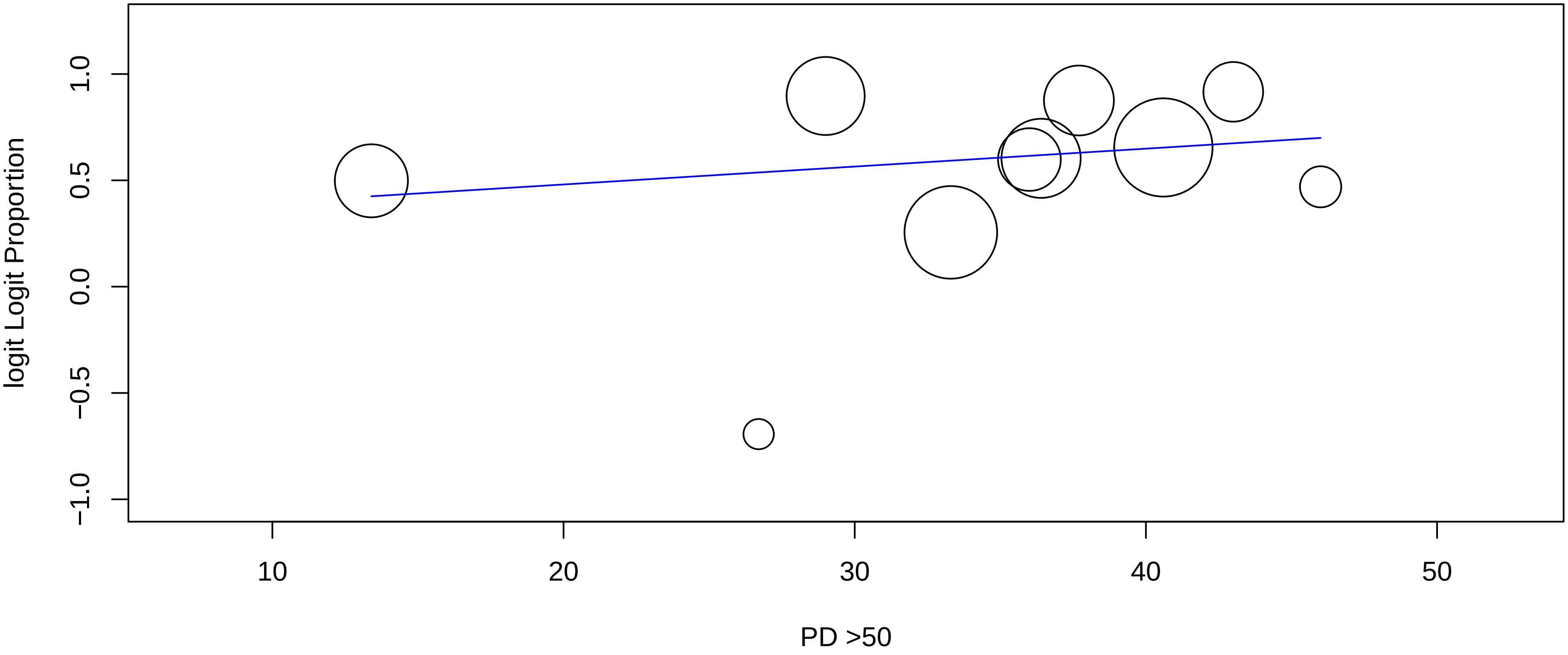

logit Logit Proportion
1.0
0.5
0.0
−0.5
−1.0
10
20
30
40
50
PD >50

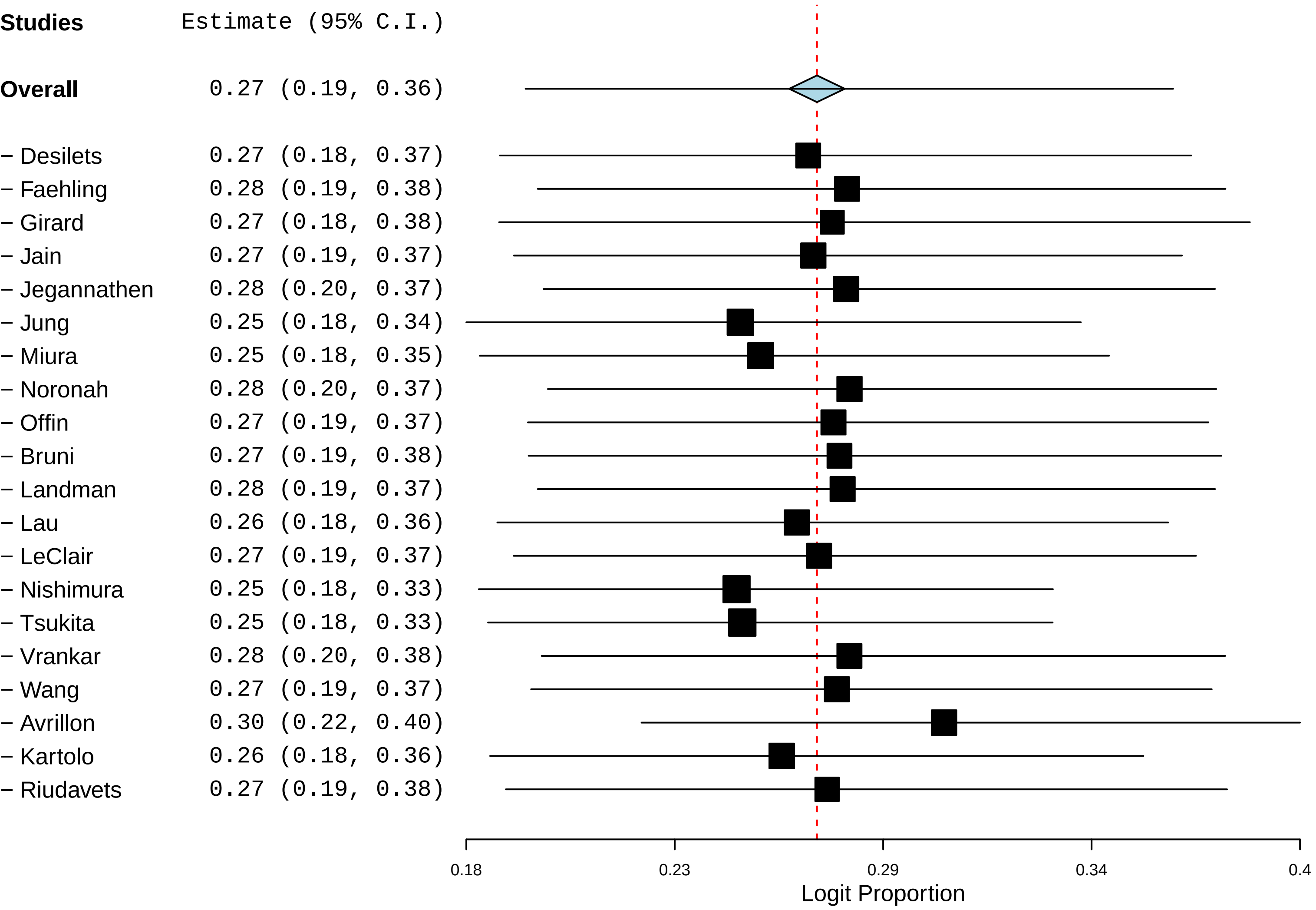

Studies
Estimate (95% C.I.)
Overall
0.27 (0.19, 0.36)
− Desilets
0.27 (0.18, 0.37)
− Faehling
0.28 (0.19, 0.38)
− Girard
0.27 (0.18, 0.38)
− Jain
0.27 (0.19, 0.37)
− Jegannathen
0.28 (0.20, 0.37)
− Jung
0.25 (0.18, 0.34)
− Miura
0.25 (0.18, 0.35)
− Noronah
0.28 (0.20, 0.37)
− Offin
0.27 (0.19, 0.37)
− Bruni
0.27 (0.19, 0.38)
− Landman
0.28 (0.19, 0.37)
− Lau
0.26 (0.18, 0.36)
− LeClair
0.27 (0.19, 0.37)
− Nishimura
0.25 (0.18, 0.33)
− Tsukita
0.25 (0.18, 0.33)
− Vrankar
0.28 (0.20, 0.38)
− Wang
0.27 (0.19, 0.37)
− Avrillon
0.30 (0.22, 0.40)
− Kartolo
0.26 (0.18, 0.36)
− Riudavets
0.27 (0.19, 0.38)
0.18
0.23
0.29
0.34
0.4
Logit Proportion

Studies	Estimate (95% C.I.)	Events/Total
Faehling	0.15 (0.09, 0.21)	19/126
Jain	0.29 (0.12, 0.45)	8/28
Jegannathen	0.11 (−0.03, 0.26)	2/18
Offin	0.19 (0.10, 0.29)	12/62
Bruni	0.17 (0.11, 0.23)	27/155
Lau	0.37 (0.26, 0.47)	30/82
LeClair	0.25 (0.16, 0.35)	21/83
Vrankar	0.14 (0.07, 0.22)	12/85
Avrillon	0.02 (0.00, 0.03)	6/399
Kartolo	0.46 (0.34, 0.58)	29/63
Riudavets	0.21 (0.17, 0.25)	68/323
**Subgroup Western (I^2=96% , P=0.00)**	**0.21 (0.12, 0.30)**	**234/1424**
Jung	0.81 (0.64, 0.98)	17/21
Miura	0.61 (0.46, 0.76)	25/41
Noronah	0.07 (−0.06, 0.19)	1/15
Landman	0.15 (0.04, 0.27)	6/39
Nishimura	0.74 (0.65, 0.84)	61/82
Tsukita	0.71 (0.62, 0.80)	76/107
Wang	0.18 (0.08, 0.28)	11/61
**Subgroup Asia (I^2=97% , P=0.00)**	**0.47 (0.23, 0.70)**	**197/366**
**Overall (I^2=98% , P=0.00)**	**0.31 (0.20, 0.42)**	**431/1790**

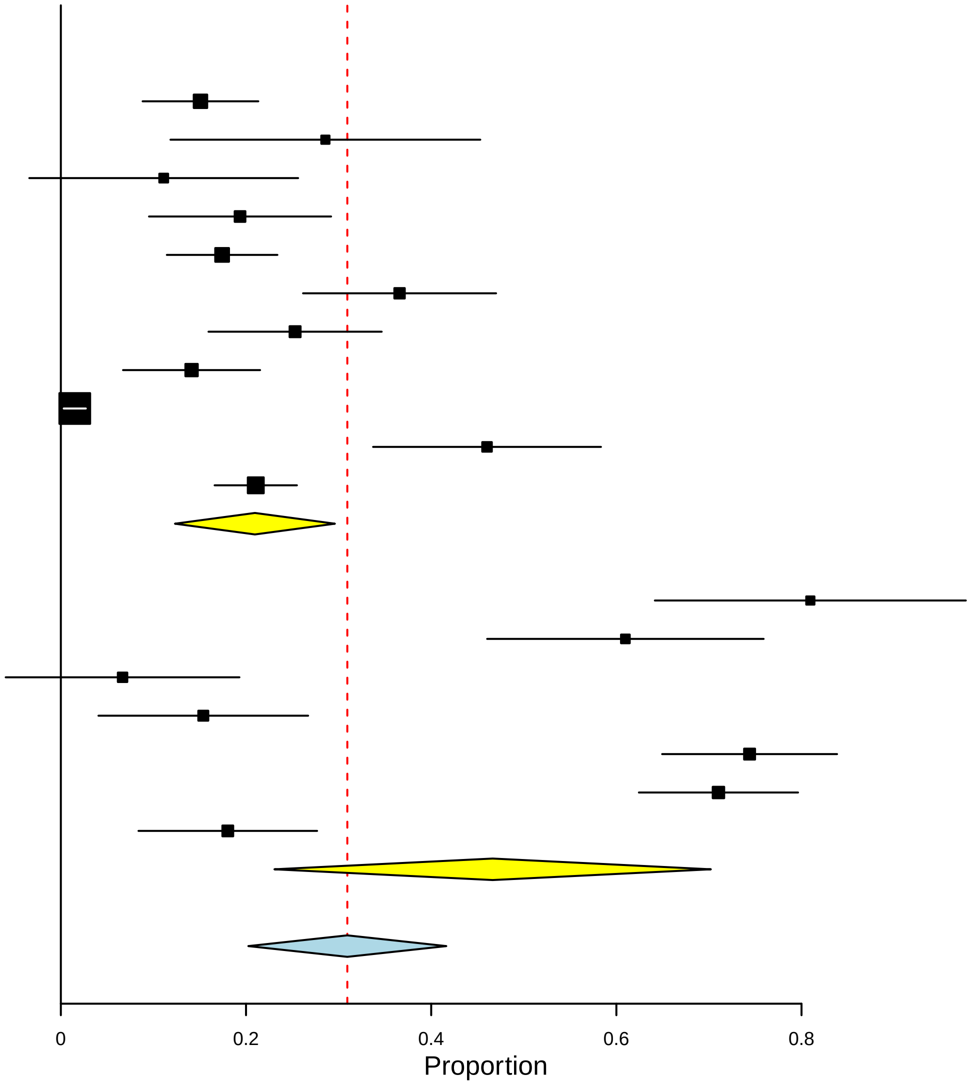

Studies	Estimate (95% C.I.)	Events/Total
Desilets	0.30 (0.23, 0.37)	44/147
Faehling	0.15 (0.09, 0.21)	19/126
Girard	0.19 (0.16, 0.21)	214/1155
Jain	0.29 (0.12, 0.45)	8/28
Jegannathen	0.11 (−0.03, 0.26)	2/18
Bruni	0.17 (0.11, 0.23)	27/155
Tsukita	0.71 (0.62, 0.80)	76/107
Wang	0.18 (0.08, 0.28)	11/61
Avrillon	0.02 (0.00, 0.03)	6/399
Kartolo	0.46 (0.34, 0.58)	29/63
Riudavets	0.21 (0.17, 0.25)	68/323
**Subgroup Multi (I^2=98% , P=0.00)**	**0.25 (0.15, 0.35)**	**504/2582**
Jung	0.81 (0.64, 0.98)	17/21
Miura	0.61 (0.46, 0.76)	25/41
Noronah	0.07 (−0.06, 0.19)	1/15
Offin	0.19 (0.10, 0.29)	12/62
Landman	0.15 (0.04, 0.27)	6/39
Lau	0.37 (0.26, 0.47)	30/82
LeClair	0.25 (0.16, 0.35)	21/83
Nishimura	0.74 (0.65, 0.84)	61/82
Vrankar	0.14 (0.07, 0.22)	12/85
**Subgroup Single (I^2=96% , P=0.00)**	**0.37 (0.20, 0.53)**	**185/510**
**Overall (I^2=98% , P=0.00)**	**0.30 (0.22, 0.38)**	**689/3092**

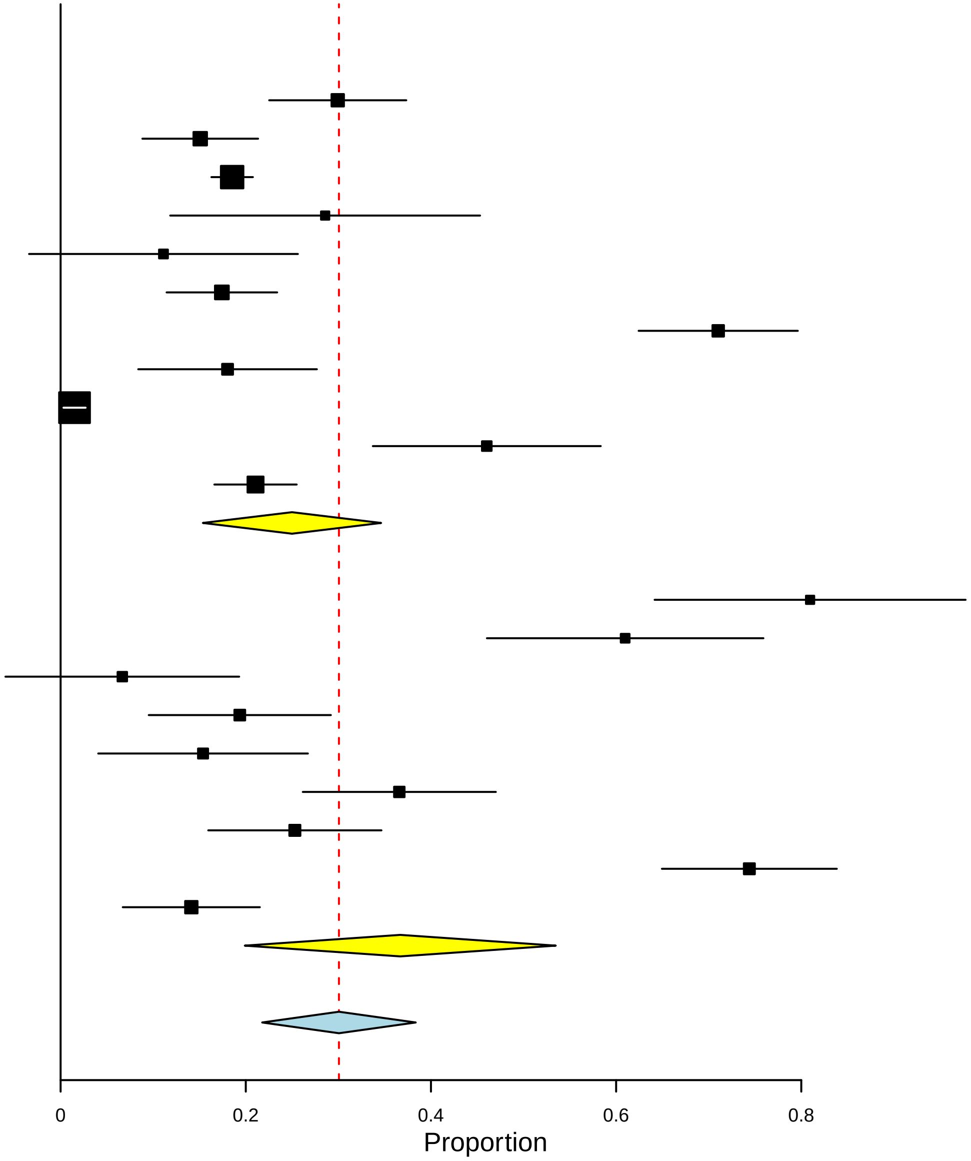

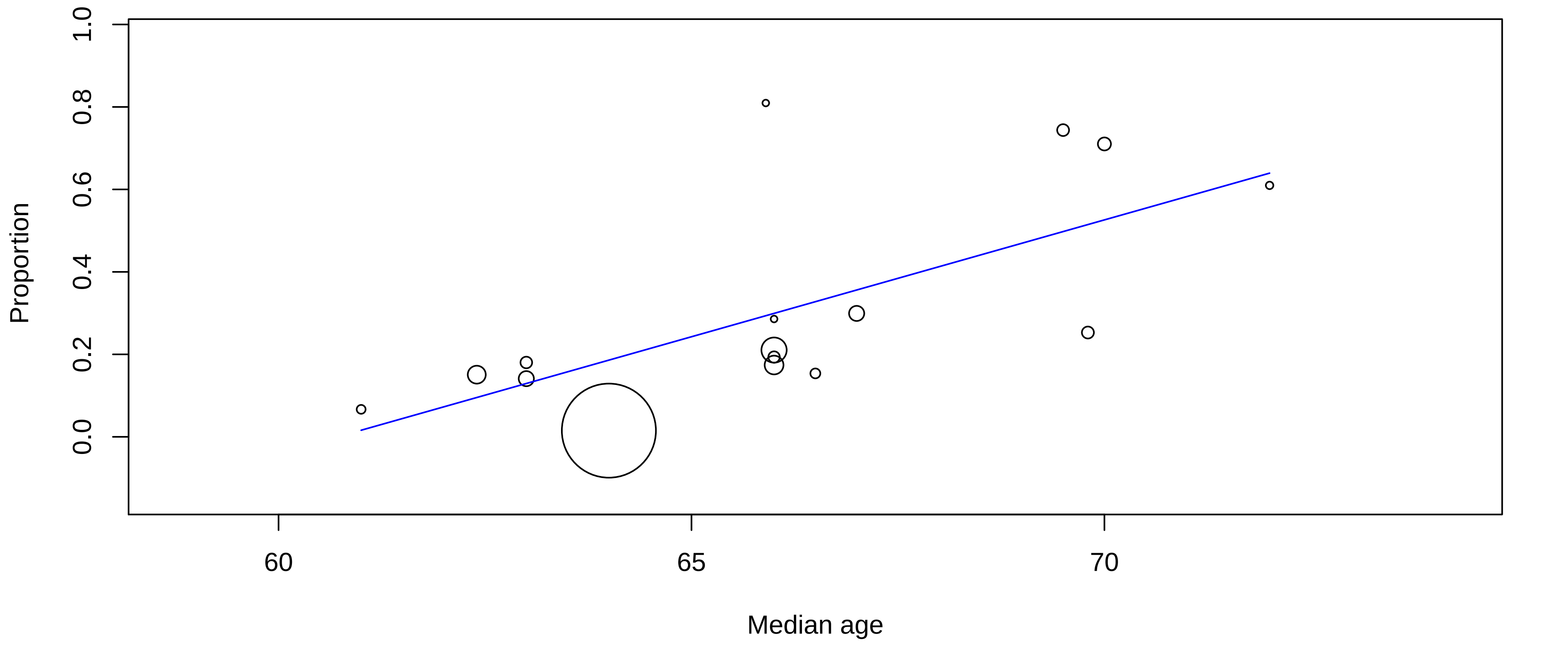

Proportion
0.0
0.2
0.4
0.6
0.8
1.0
60
65
70
Median age

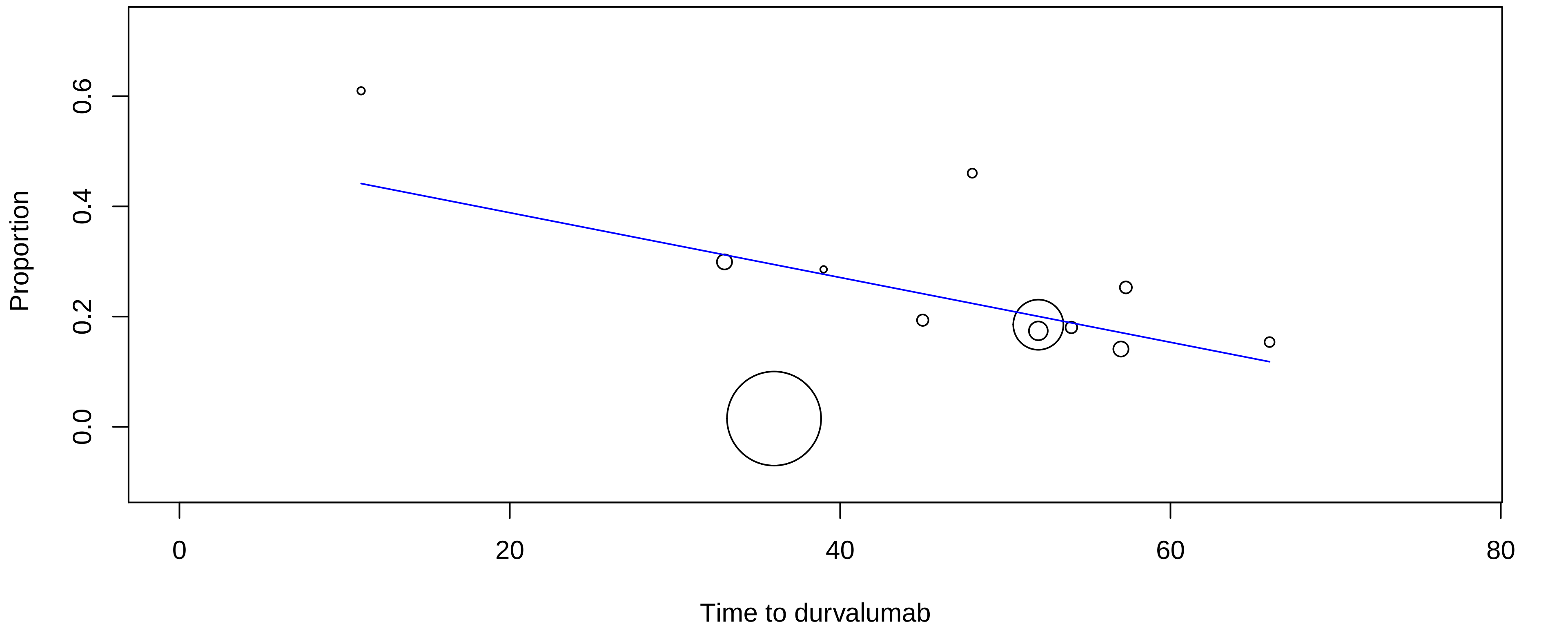

Proportion
0.0
0.2
0.4
0.6
0
20
40
60
80
Time to durvalumab

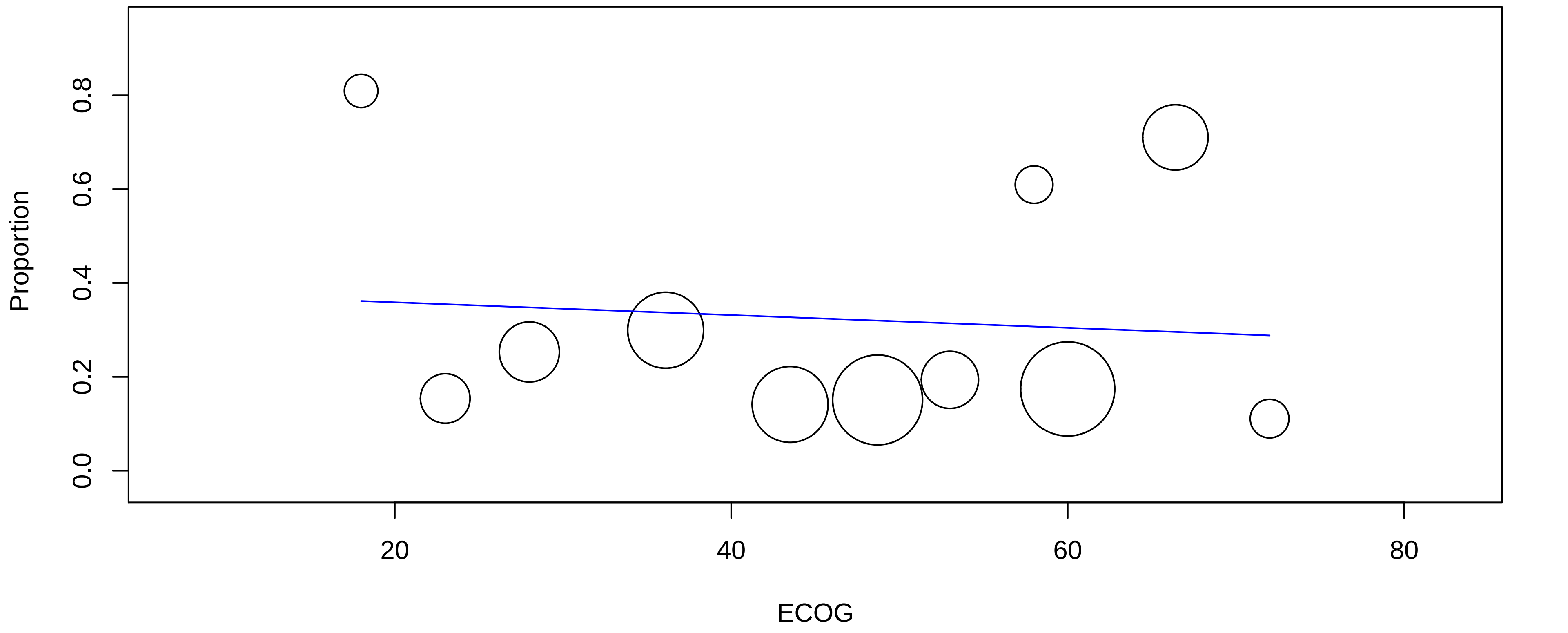
Proportion
0.0
0.2
0.4
0.6
0.8
20
40
60
80
ECOG

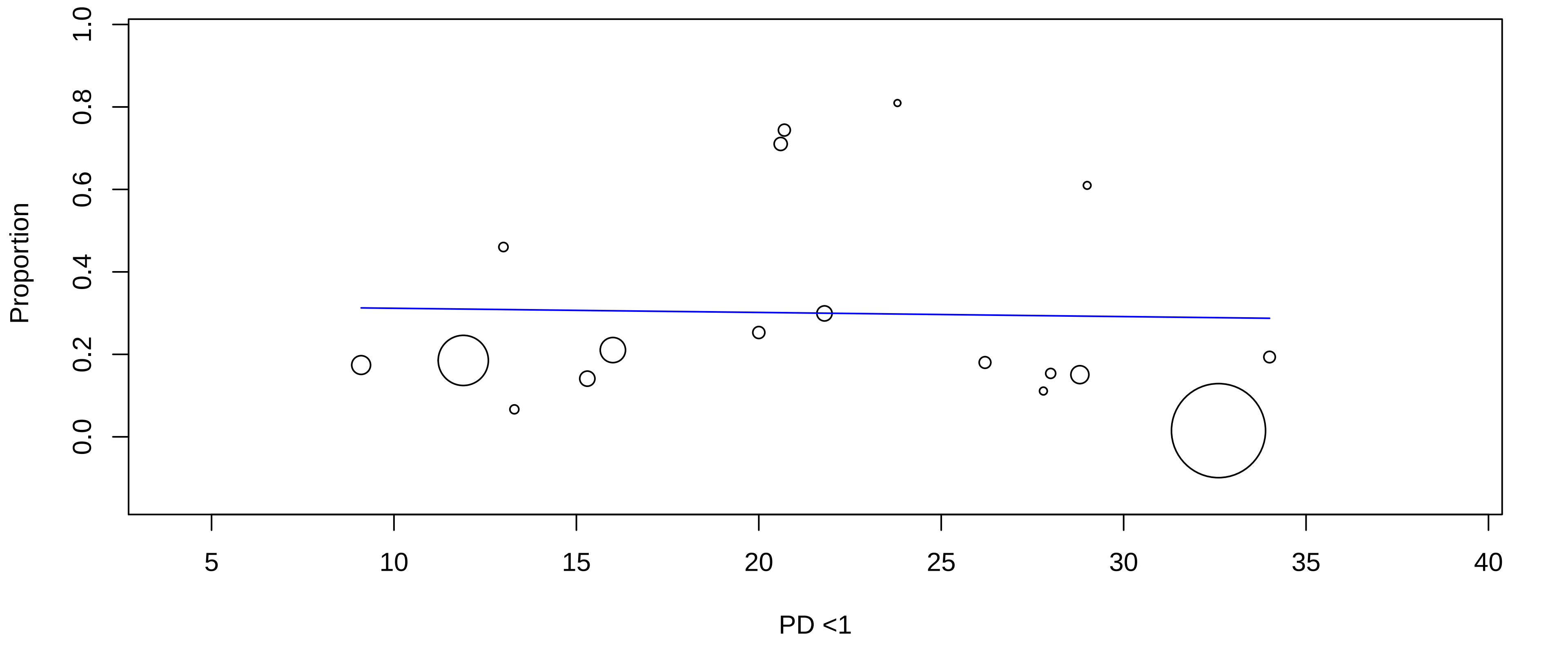

1.0
0.8
0.6
0.4
0.2
0.0
Proportion
5
10
15
20
25
30
35
40
PD <1

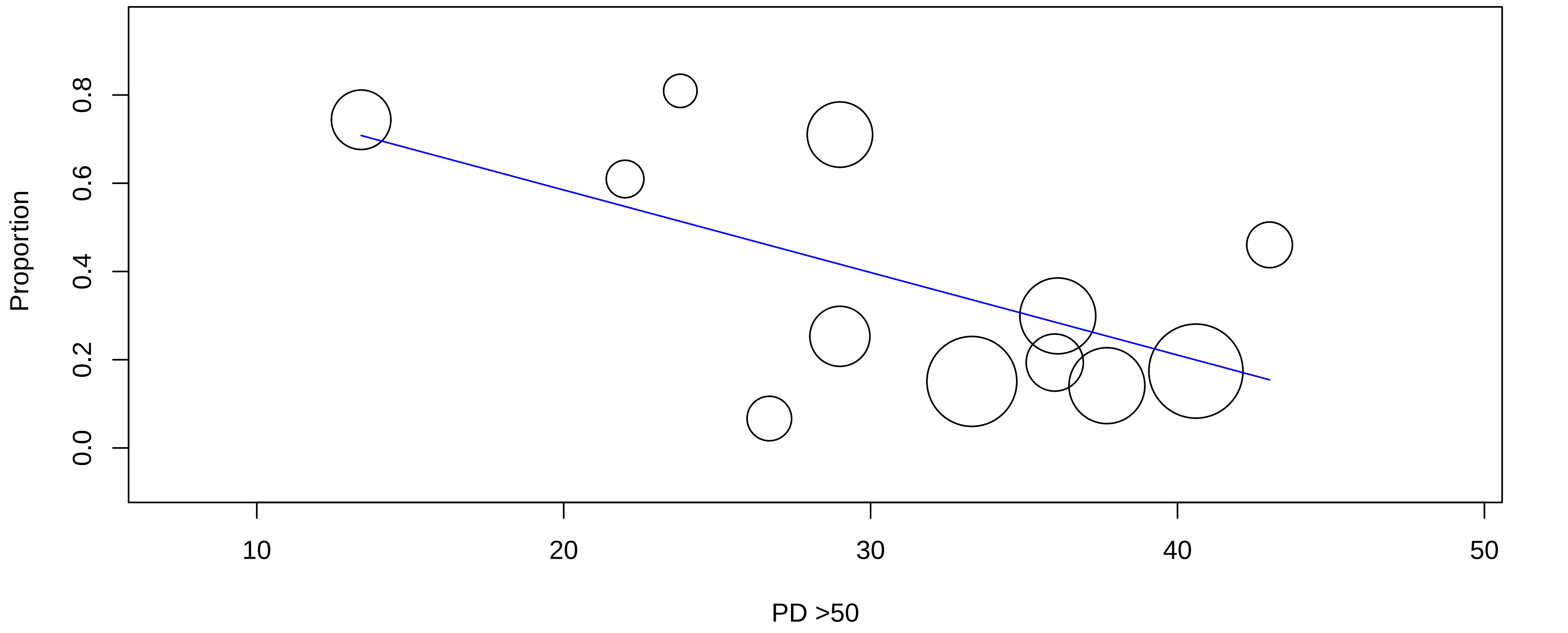
Proportion
0.0
0.2
0.4
0.6
0.8
10
20
30
40
50
PD >50
